# Unseen overlap between fishing vessels and top predators in the northeast Pacific

**DOI:** 10.1126/sciadv.adl5528

**Published:** 2024-03-06

**Authors:** Heather Welch, Tyler Clavelle, Timothy D. White, Megan A. Cimino, David Kroodsma, Elliott L. Hazen

**Affiliations:** ^1^ Institute of Marine Science, University of California, Santa Cruz, Santa Cruz, CA, USA.; ^2^ Environmental Research Division, Southwest Fisheries Science Center, Monterey, CA, USA.; ^3^ Global Fishing Watch, Washington, DC, USA.; ^4^ Hopkins Marine Station, Stanford University, Pacific Grove, CA, USA.

## Abstract

Accurate assessments of human-wildlife risk associated with industrial fishing are critical for the conservation of marine top predators. Automatic Identification System (AIS) data provide a means of mapping fishing and estimating human-wildlife risk; however, risk can be obscured by gaps in the AIS record due to technical issues and intentional disabling. We assessed the extent to which unseen fishing vessel activity due to AIS gaps obscured estimates of overlap between fishing vessel activity and 14 marine predators including sharks, tunas, mammals, seabirds, and critically endangered leatherback turtles. Among vessels equipped with AIS in the northeast Pacific, up to 24% of total predator overlap with fishing vessel activity was unseen, and up to 36% was unseen for some individual species. Waters near 10°N had high unseen overlap with sharks yet low reported shark catch, revealing potential discrepancies in self-reported datasets. Accounting for unseen fishing vessel activity illuminates hidden human-wildlife risk, demonstrating challenges and solutions for transparent and sustainable marine fisheries.

## Teaser

Gaps in Automatic Identification System data obscure human-wildlife risk at sea, masking the urgency of risk mitigation.

## INTRODUCTION

Many populations of marine predators are in decline due to interactions with industrial fisheries, including overexploitation of target catch, bycatch, and ship strike (*1*–*5*). Understanding the magnitude and locations of these adverse interactions is critical for predator conservation and management, yet a paucity of data exists due to the remoteness and vastness of the oceans (*2*). The Automatic Identification System (AIS) is a vessel tracking system designed to prevent vessel collisions but has become an emergent tool to understand the behaviors and footprints of the world’s fishing fleets (*6*, *7*). AIS data are frequently combined with animal movement data or species distribution models to provide critical insights into human-wildlife risk that is unobserved or unreported (*8*–*15*).

However, the utility of AIS data as a tool for identifying human-wildlife risk is impeded by gaps in the AIS record (*16*). AIS gaps occur due to technical phenomena such as poor satellite coverage and signal interference in crowded areas. In addition, vessels legally and illegally disable their AIS devices to obscure the locations of high quality fishing grounds, vessel positions in dangerous waters prone to piracy, or unauthorized geopolitical border crossings and transshipment events (*16*). Thus, vessel activity observable in the AIS record represents only a portion of activity by vessels equipped with AIS devices. By extension, assessments of human-wildlife risk that are based on AIS are likely underestimated, potentially masking the urgency of human-wildlife risk mitigation.

To understand how missing data in the AIS record affects assessments of human-wildlife risk, we harnessed a recently released global dataset of unseen fishing vessel activity by vessels equipped with AIS (*16*). This dataset provided the first global view of unseen activity due to gaps caused by technical phenomena and intentional disabling, affording insights into locations and fleets where the utility of AIS as a monitoring tool is compromised. The unseen fishing vessel activity dataset provides an unprecedented opportunity to understand the proportion of human-wildlife risk that is observable in the AIS record and to reveal hidden hot spots of risk.

Unseen (*16*) and observed (*6*) fishing vessel activity were overlaid with the predicted distributions of 14 top predator species to quantify and map human-wildlife risk (i.e., overlap) from 2017 to 2022. Species distribution models (*17*) were derived from extensive telemetry datasets (*18*–*20*) for tunas, sharks, seabirds, mammals, and leatherback turtles. Of the 14 predators investigated, nine are listed as near-threatened, vulnerable, endangered, or critically endangered by the International Union for the Conservation of Nature (IUCN), with blue whales and leatherback turtles also listed endangered under the Endangered Species Act (ESA) (table S1). These species have a variety of interactions with fisheries, including, target catch (the primary targets of fisheries effort: tunas), nontarget catch (incidentally caught while fishing for target species but landed and sold: blue, mako, and salmon sharks), bycatch (incidentally caught while fishing but not landed or sold: seabirds, mammals, leatherback turtles, and white sharks), ship strike, and entanglement in fishing gear (blue whales and leatherback turtles) (*2*, *4*, *8*, *9*, *21*–*23*).

The northeast Pacific is a biodiversity hot spot and critical foraging ground for these top predators, many of which are of high economic or ecological value (*18*). Many predators are highly mobile, undertaking large-scale migrations from across the broader Pacific to reach the California Current System (*24*). These broad-scale movements bring predators into contact with fishing fleets from multiple nations across several different management regimes (*8*). This complex network of threat and governance makes AIS data a critical tool to evaluate interactions between highly migratory predators and fishing vessels. Other vessel tracking datasets such as national Vessel Monitoring System (VMS) data are infrequently shared between nations, and international self-reported catch and effort data provided to Regional Fisheries Management Organizations (RFMOs) are often biased toward nations with high levels of transparency (*25*). Understanding where human-wildlife risk may be obscured by unseen fishing vessel activity increases the utility of AIS data as a tool to promote sustainable and transparent fisheries.

## RESULTS

### Unseen overlap in the Northeast Pacific

**Fig. 1.**
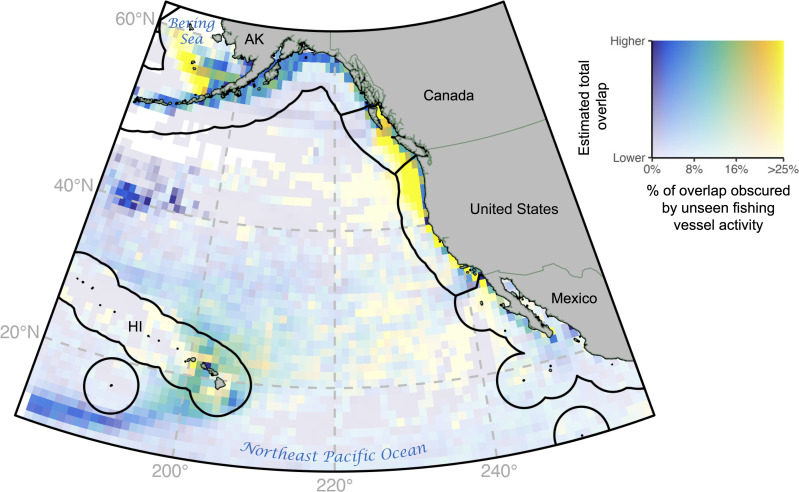
Estimated total and unseen overlap of fishing vessels and top predators in the northeast Pacific from 2017 to 2022. Estimated total overlap (*y* axis) and the percentage of this overlap obscured by unseen fishing vessel activity (*x* axis). Black lines indicate Exclusive Economic Zone (EEZ) boundaries. Top predators include target species (yellowfin, albacore, and bluefin tunas), nontarget species (blue, mako, and salmon sharks), and bycatch species (blue whales, elephant seals, California sea lions, leatherback turtles, white sharks, Laysan and black-footed albatrosses, and sooty shearwaters). State codes: Alaska (AK) and Hawaii (HI), both belonging to the United States.

Across the study period, 1900 to 5500 hours (6 to 19%) of fishing vessel activity were obscured by AIS gap events—periods of missing AIS data due to intentional AIS disabling and technical issues such as low satellite coverage (fig. S1). Gap events were most common in drifting longlines and trawlers, and vessels with gap events ranged from 8 to 110 m in length (mean = 25 m) and from 4 to 5770 gross tons (gt) in weight (mean = 202 gt) (fig. S2). Accounting for this unseen activity increased the total overlap between top predators and fishing vessel activity by 8 to 24%. These ranges represent AIS gaps longer than 2 weeks excluded and included to capture the lower and upper estimation bounds, respectively (see Materials and Methods). Hot spots of unseen overlap—locations with high estimated total overlap and a high percentage of this overlap obscured by unseen fishing vessel activity—were located near Alaska in the Bering Sea and along the North American west coast (Fig. 1 and fig. S3).

**Fig. 2.**
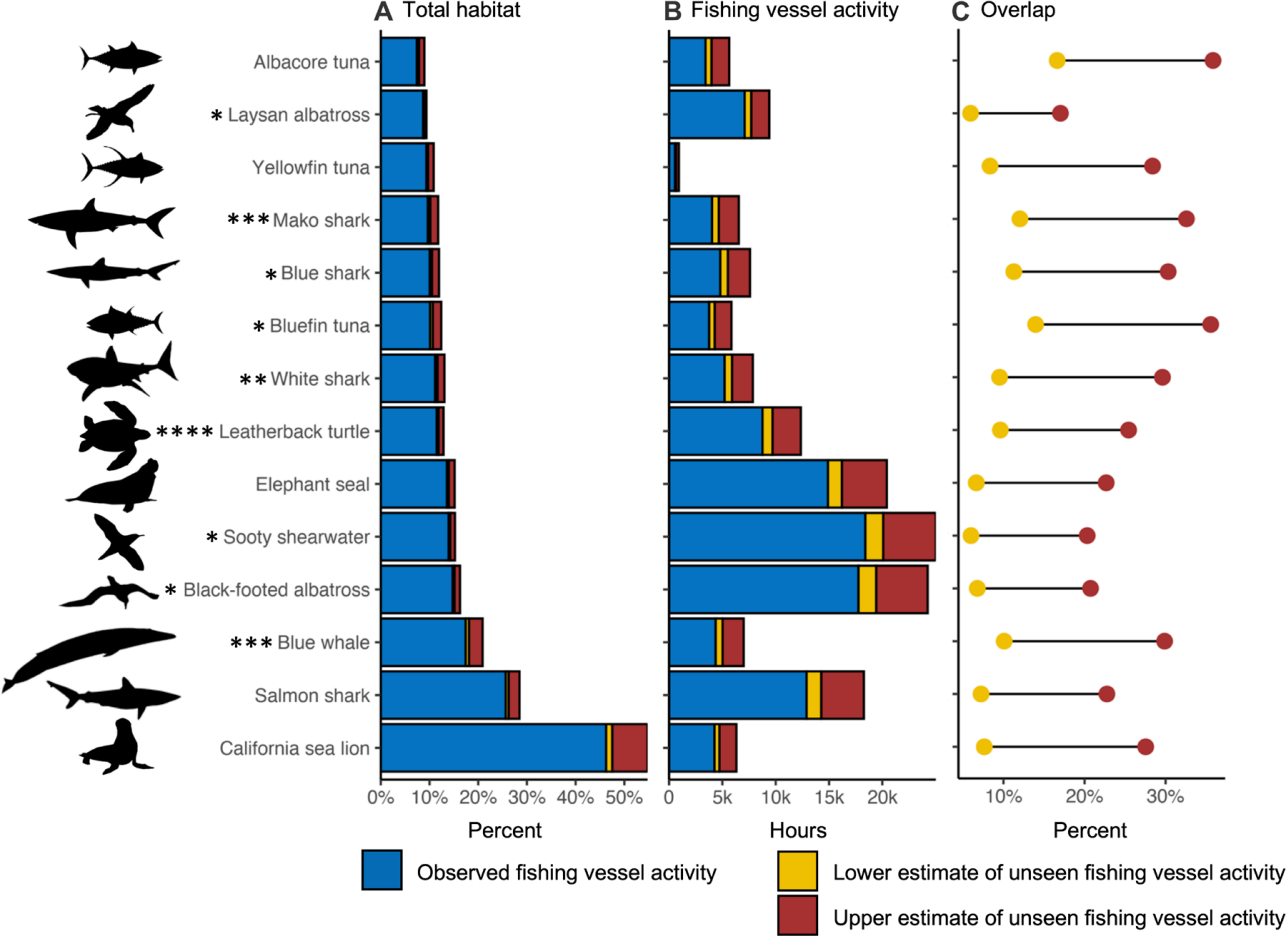
Relationship between species habitats, fishing vessel activity, and overlap. Overlap is a function of the amount of species habitat and the intensity (hours) of fishing vessel activity that coincide in space and time. Because overlap is unitless and difficult to interpret in absolute terms, the base components of overlap are also presented: (**A**) the percent of each species’ total habitat coinciding with fishing vessel activity (ignoring the intensity of fishing vessel activity) and (**B**) the intensity of fishing vessel activity coinciding with each species’ habitat (ignoring the amount of species habitat). (**C**) Percent of overlap between species and fishing vessel activity obscured by unseen fishing vessel activity. Lower and upper estimation bounds of unseen fishing vessel activity in (A) to (C) represent gaps longer than 2 weeks excluded and included, respectively. Population status is represented as: *, for near threatened; **, for vulnerable; ***, for endangered; ****, for critically endangered; all other species are of least concern.

The percent of species’ total habitat coinciding with observed fishing vessel activity ranged from 7 to 46% (blue bars in Fig. 2A), and the intensity of observed fishing vessel activity coinciding with each species’ habitat ranged from 570 to 18,400 hours (blue bars in Fig. 2B). When unseen fishing vessel activity was accounted for, the percent of species total habitat coinciding with fishing vessel activity increased by up to 7% (red bars in Fig. 2A), and the intensity of fishing vessel activity coinciding with each species’ habitat increased by up to 4940 hours (red bars in Fig. 2B). Accounting for unseen fishing vessel activity increased overlap by up to 36% for albacore and bluefin tunas and by up to 33 and 30% for mako and blue sharks, respectively (red points in Fig. 2C).

### Case study I: Unseen bycatch risk

**Fig. 3.**
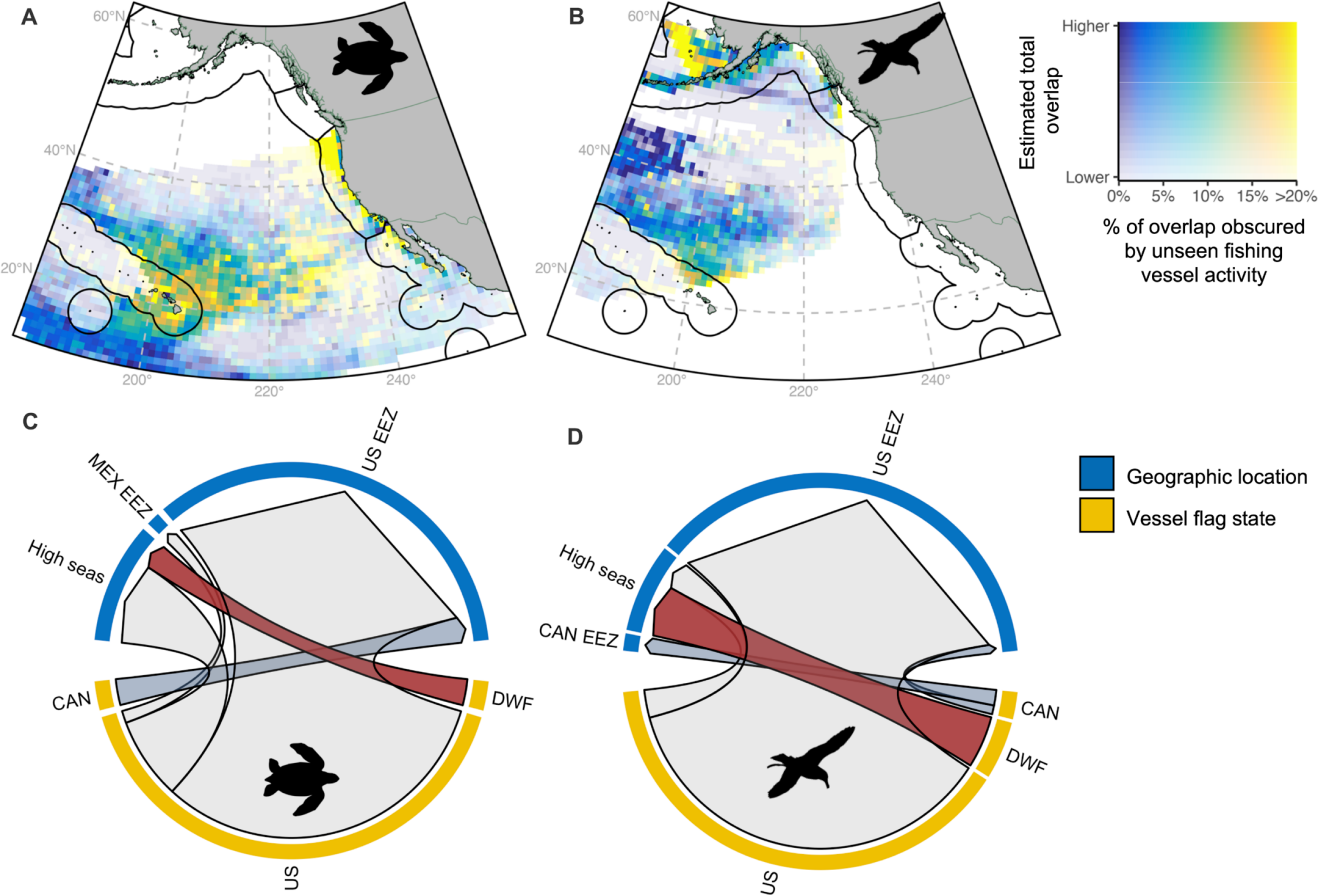
Overlap of unseen fishing vessel activity with two bycatch species. Estimated total overlap and the percentage of this overlap obscured by unseen fishing vessel activity for (**A**) critically endangered leatherback turtles and (**B**) near-threatened Laysan albatross. Unseen overlap by vessel flag state and geographic location for (**C**) leatherback turtles and (**D**) Laysan albatross. Roughly two-thirds of unseen Laysan albatross overlap in the high seas occurred with distant water fleets (DWF) and the remainder occurred with US flagged vessels. Prediction domains [(A) and (B)] for each species are spatially constrained to the extent of the data used to build the models; black lines indicate EEZ boundaries. Country codes: CAN: Canada; US: United States; MEX: Mexico.

Using two bycatch species as a case study, we explored how unseen overlap varied by flag state and geographic location (Fig. 3). Leatherback turtles and Laysan albatross are endangered and near-threatened, respectively, and are both affected by industrial fisheries: Between 2009 and 2015, the U.S. reported 2190 leatherbacks and 2320 Laysan albatross bycaught across its fisheries (*23*).

Most of the unseen overlap with both species occurred with U.S. flagged vessels fishing in U.S. waters (leatherbacks: Hawaii, Oregon, and Washington; Laysan albatross: Alaska). High values for the United States are expected given this study’s focal region: In the northeast Pacific, more than 80% of observed fishing vessel activity and 82% of observed overlap occurs with U.S.-flagged fishing vessels (fig. S4). Seven percent of unseen overlap with leatherbacks in U.S. waters occurred with Canadian-flagged fishing vessels (Fig. 3C). Distant water fleets (Vanuatu, Chinese Taipei, China, and others) were responsible for 24 and 67% of unseen overlap with leatherbacks and Laysan albatross in the high seas, respectively (Fig. 3, C and D).

### Case study II: Potential reporting discrepancies

**Fig. 4.**
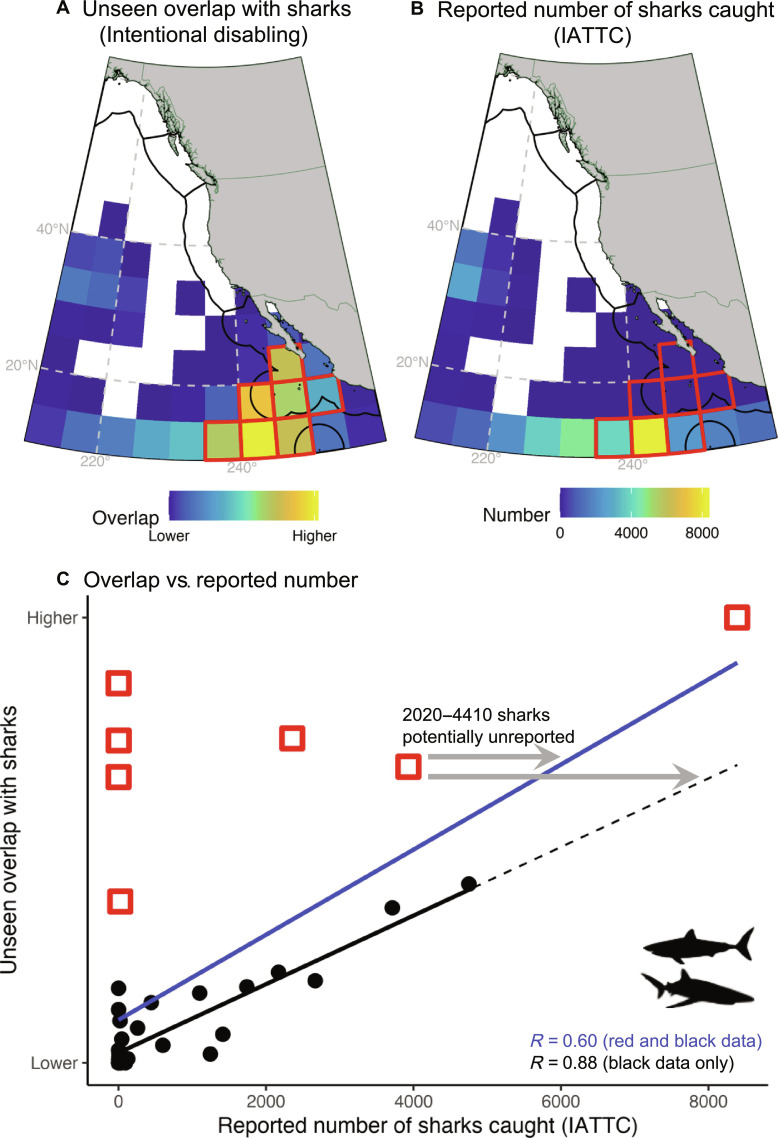
Potential shark reporting discrepancies. (**A**) Unseen overlap between sharks and vessels with intentionally disabled AIS devices. (**B**) Shark catch reported to the IATTC. (**C**) Relationship between unseen overlap (A) and reported shark catch (B). Line of best fit to full dataset in blue; black line shows the line of best fit to a partial dataset excluding seven anomalous areas with high unseen overlap and low reported shark catch [red squares in (A) to (C)]. Gray arrows indicate the range of potentially unreported shark catch based on lines of best fit; Pearson’s correlation coefficients (*R*) in blue and black text are reported for full and partial datasets, respectively. All panels use data on blue and mako sharks from 2017 to 2021 for non-U.S.–flagged fishing vessels fishing with tuna purse seines and longlines.

While most of the unseen fishing vessel activity was due to technical issues, e.g., low satellite coverage, 11 to 16% were caused by vessels intentionally disabling their AIS devices. Intentional disabling events were most common in drifting longlines and trawlers, and vessels with gap events ranged from 11 to 109 m in length (mean = 34 m) and from 14 to 5770 gt in weight (mean = 420 gt) (fig. S5). We explored the relationship between unseen overlap of sharks with intentionally nonbroadcasting fishing vessels and shark catch reported to the Inter-American Tropical Tuna Commission (IATTC; Fig. 4).

Agreement between unseen overlap and reported catch was high (*R* = 0.60; Fig. 4C); this is to be expected, as vessels frequently disable their AIS devices in high-quality fishing grounds to reduce competition. However, there were seven anomalous areas with high unseen overlap and low reported catch (red colors in Fig. 4, A to C). These areas may be locations where vessels are catching sharks but not reporting this catch to the IATTC. When these anomalous areas were removed, the agreement between unseen overlap and reported catch increased (*R* = 0.88). Total shark catches across areas with high unseen overlap and low reported catch would increase by 30,000 to 48,000 sharks, which was determined using the line of best fit and summing the estimated number of unreported sharks (gray arrows, Fig. 4C) across anomalous areas. Nearly all of the shark catch reported to the IATTC in anomalous areas was reported by Chinese-flagged fishing vessels, with only 0.03% reported by Mexican-flagged fishing vessels (fig. S6, A and D). However, 90% of unseen overlap in these areas occurred with Mexican-flagged fishing vessels, with the remaining 10% occurring with Chinese-flagged fishing vessels.

## DISCUSSION

We investigated how gaps in the AIS record obscure human-wildlife risk at sea. Previously, periods of missing AIS data limited the utility of AIS as a management and conservation tool by creating blindspots in our estimates of fishing vessel activity and species overlap. Here, we illuminate these blindspots by combining a recently released dataset of unseen fishing vessel activity (*16*) with observed fishing vessel activity (*6*) and the predicted distributions of 14 top predators (*17*). The unseen fishing vessel activity revealed up to an additional 19% (5500 hours) of activity from 2017 to 2022 by fishing vessels equipped with AIS, allowing for additional insight on human-wildlife risk in the northeast Pacific.

Accounting for unseen fishing vessel activity increased the total overlap with predators by up to 24%, indicating that previous overlap assessments using observed AIS are likely underestimates. Prior work in the northeast Pacific on the seven shark and tuna species investigated here found similar magnitudes of overlap with observed AIS (*8*, *14*). Across these species, overlap increased by 23 to 36% when unseen fishing vessel activity was accounted for (Fig. 2C). Overlap with black-footed and Laysan albatross increased by 21 and 17%, respectively, suggesting previous assessments for this region may underestimate interaction risk (*11*). In particular, overlap is likely underestimated in U.S. waters near the U.S. west coast and Alaska, where most of the unseen overlap occurs for each species aside from yellowfin tuna (Fig. 1 and figs. S2 and S6). Prior work on blue whale ship strike along the U.S. west coast has focused on large tanker, passenger, and cargo vessels (*9*, *15*, *26*), but ships of all sizes and types can strike whales (*27*, *28*). Blue whale overlap increased by 31% when unseen fishing vessel activity was accounted for, allowing for additional inferences on the intensity of near-shore ship strike risk.

Countries have national data streams for tracking their fishing vessels beyond AIS, and when a vessel disappears from the AIS record, its activities are likely still visible to its flag state (*29*, *30*). For example, the United States monitors its own fishing effort using VMS, logbook, and observer data. However, these data are confidential and are rarely shared across flag states. While RFMO data are compiled across flag states, bycatch data are only representative of small portions of fishing effort. RFMO bycatch data for turtles, seabirds, and mammals are primarily based on observer data, but observers are required for only 5% of longline vessels in the northeast Pacific (*31*, *32*). This level of coverage has been found insufficient for estimating total catch for data-rich target species, e.g., tunas (*31*). Much higher coverage would be required to estimate catches of species caught less frequently such as seabirds and turtles (*33*).

AIS-based overlap has potential to complement RFMO data (*6*, *34*, *35*) and may provide a more holistic view of human-wildlife risk for data-poor species, particularly in locations fished by multiple flag states such as the high seas. While the United States was responsible for the vast majority of observed fishing vessel activity across the northeast Pacific (fig. S4), in the high seas, it comprised only 53 and 13% of observed overlap with leatherback turtles and Laysan albatross, respectively (fig. S7). The remaining high seas observed overlap with both species came from distant water fleets such as China, Japan, and Chinese Taipei, and these fleets accounted for 24 and 67% of unseen overlap with leatherback turtles and Laysan albatross, respectively (Fig. 3 and fig. S8). Information gaps have hindered large-scale estimates of marine turtle and seabird bycatch (*1*, *2*, *36*); accounting for unseen fishing vessel activity provides a missing link toward quantifying threats posed by fisheries to species that are underrepresented in RFMO datasets.

Nations are required to self-report fleet-wide and spatially resolved catch and effort information (*37*). However, self-reported data provided to RFMOs are typically biased toward nations with higher transparency (*25*), and underreporting or nonreporting is common (*35*, *38*–*40*). The only fleet that reports its total catches of sharks to the IATTC is the U.S. longline fleet (*25*). We assessed the ability of unseen overlap due to intentional disabling to identify data gaps in self-reported blue and mako shark catch provided to the IATTC by non-U.S. fleets (Fig. 4). Several areas located near 10°N and within the Mexican Exclusive Economic Zone (EEZ) had high unseen overlap and relatively low reported shark catch. On the basis of the magnitude of unseen overlap with intentional disabling in these anomalous areas, our results suggest that between 30,000 and 48,000 more sharks (above 35,332 sharks reported) are potentially being caught.

While intentional disabling can occur for a variety of legal reasons including to reduce competition with other fishing vessels, it is also done to obscure nefarious activities from oversight (*16*). A portion of vessels active in areas with high unseen overlap and low reported catch may be disabling their AIS devices to obscure catch that they do not plan to report. The majority (90%) of unseen overlap in these areas occurred with Mexican-flagged fishing vessels; however, Mexico reported only four sharks caught to the IATTC. Archival data show that Mexico historically caught blue and mako sharks in these waters (fig. S6B), but spatially resolved longline shark catch has not been reported by Mexico since the 1980s. Notably, Mexico reports substantial tuna and billfish catch in these areas during our study period (fig. S6C), indicating that Mexican-flagged fishing vessels are active in these areas and are likely catching sharks alongside targeted fish. The remaining 10% of unseen overlap with intentional disabling occurred with Chinese-flagged fishing vessels. While China reported 14,700 sharks in these anomalous areas during our study period, we estimate that actual catch may be 1000 to 2600 individuals higher. Self-reported catch data from China are frequently distorted (*41*), and China’s distant water fleet is estimated to underreport catch by an order of magnitude (*38*).

Estimates of catch based on the relationship between unseen overlap with intentional disabling and reported catch likely have high uncertainty. There is high uncertainty in RFMO data, which suffer from underreporting, nonreporting, lack of resolution on gear type and taxa, and noncomprehensive requirements for reporting catch fate (e.g., retained or discarded) (*35*). Despite requirements to report spatially resolved catch data (*37*), catch is often reported as nonspatial fleetwide summaries (*35*). Furthermore, there is perceived ambiguity regarding reporting requirements for sharks among IATTC member countries. Intentional disabling can not only occur to obscure fishing activity but also occurs to obscure other behaviors such as transiting and transshipping (*16*). Thus, our estimates of potential reporting discrepancies are likely precautionary overestimates. Furthermore, while overlap provides insights as to where and when species and fisheries coincide in space and time, overlap does not necessarily result in interaction. Our overlap metric does not consider the vertical dimension of overlap, nor does it consider fishing effort (e.g., number of hooks) or animal abundance. Last, our study is limited to fishing vessels equipped with AIS, which is estimated as 52 to 85% of fishing vessels over 24 m in length but likely includes the majority of longline vessels within RFMOs (*42*, *43*). While accounting for unseen activity may improve assessments of AIS-based overlap, it does not capture overlap with vessels not equipped with AIS and thus our assessments are likely underestimated. Recent advances in satellite mapping provide a promising avenue for tracking fishing vessels not equipped with AIS (*44*) and refining estimates of human-wildlife risk.

### Toward transparent and sustainable fisheries

Unseen fishing vessel activity promotes fisheries transparency by increasing the availability and flow of information on human activities at sea. When unseen fishing vessel activity is integrated with animal movement data to produce unseen overlap, it provides information that could be applied to support each of the three pillars of fisheries sustainability: science, management, and enforcement (*45*, *46*).

### Science

Overlap is frequently used to assess human-wildlife risk at sea: catch and bycatch risk (*11*, *12*, *14*, *47*–*49*), ship strike risk (*9*, *13*, *15*, *26*), non-native species introduction risk (*50*), and noise pollution risk (*51*, *52*). The inclusion of unseen overlap in such metrics improves our scientific assessment of the magnitudes, locations, and parties involved in human-wildlife risk (e.g., “the Case study I” section).

### Management

Improved assessment of the magnitudes of risk could affect species’ stock assessments or mortality estimates (*53*, *54*), as well as species’ statuses and management requirements under regulatory conservation bodies such as the IUCN, the ESA, the Convention on International Trade in Endangered Species of Wild Fauna and Flora or the Convention on the Conservation of Migratory Species of Wild Animals. Improved assessment of the locations of human-wildlife risk could be used to evaluate outcomes of Marine Protected Areas (MPAs) such as fisheries effort displacement (*55*) and stock spillover (*34*) or to identify management gaps (*34*, *56*) and cite new MPAs (*57*, *58*). Improved assessment of the parties involved in human-wildlife risk could help identify fleets for which additional regulations such as gear modifications are needed (*14*).

### Enforcement

Intentional disabling has proven to be an actionable tool for detecting and charging vessels engaged in illegal, unreported, and unregulated (IUU) fishing activities (*59*, *60*). Unseen overlap with intentional disabling could be used to infer the species that vessels are fishing for in unauthorized locations (*10*, *16*, *61*) and subsequently transferring during unregulated transshipments (*16*, *56*). Unseen overlap with intentional disabling could be compared against fisheries dependent datasets to identify potential reporting discrepancies (e.g., the “Case study II” section) and noncompliance (*42*). These types of information could be used to position at sea or airborne enforcement, to guide port-based inspections, to schedule optical satellite passes, and to support IUU fishing investigations and sanctions.

Although considerable strides have been made in recent years to map environmental (*62*), biological (*63*), and industrial (*6*, *61*) ocean conditions, our oceans remain data poor. Observational data are frequently only representative of a small proportion or area of the feature being quantified or mapped. The remainder must be estimated by scaling or interpolation to achieve full coverage: global bycatch rates and seafloor topography are estimated from 5 and 20% observed coverage, respectively (*64*, *65*). Global coverage of our oceans is particularly timely: the Biodiversity Beyond National Jurisdictions agreement was ratified in March 2023, and the ongoing 30x30 Initiative was launched in 2021. Both mandates will require high coverage information on species and their threats to ensure the conservation and sustainable use of marine resources in the high seas and protect 30% of global waters by 2030, respectively. The AIS gaps dataset is both public and global (*16*), making our methodology rapidly transferable to other regions, for which species distribution models exist. Accounting for unseen fishing vessel activity due to AIS gaps provides a large-scale, systematic approach for scaling estimates of human-wildlife risk at sea beyond those observable on AIS, enhancing our understanding of where, when, and with whom risk occurs.

## MATERIALS AND METHODS

This study quantified unseen overlap between fishing vessels and top predators in the northeast Pacific. Unseen fishing activity data from 2017 to 2019 were produced by Welch *et al.* (*16*) and extended through 2022 following the methodology in the aforementioned study. Top predator distributions from 2017 to 2020 were produced by Welch *et al.* (*17*) and extended through 2022 following the same methodology. All metrics of overlap were calculated by this present study via the methods described below.

### Data

#### Species distribution data

Boosted regression tree models (*66*) were built using extensive telemetry datasets (*18*–*20*) combined with daily dynamic environmental data from Copernicus Marine Environmental Monitoring Service (CMEMS). Species included blue (*Prionace glauca*), mako (*Isurus oxyrinchus*), salmon (*Lamna ditropis*), and white (*Carcharodon carcharias*) sharks; albacore (*Thunnus alalunga*), yellowfin (*Thunnus albacares*), and bluefin (*T. orientalis*) tunas; black-footed (*Phoebastria nigripes*) and Laysan (*Phoebastria immutabilis*) albatrosses; sooty shearwaters (*Ardenna grisea*); blue whales (*Balaenoptera musculus*); California sea lions (*Zalophus californianus*); elephant seals (*Mirounga angustirostris*); and leatherback turtles (*Dermochelys coriacea*). Daily environmental data from CMEMS included primary productivity averaged across the upper 200 m of the water column, oxygen concentration at 200 m, sea surface temperature and its spatial SD, sea level anomaly, eddy kinetic energy, mixed layer depth, and chlorophyll-a. Additional model covariates included bathymetry, day of year, and rugosity—a proxy for seabed complexity calculated as the spatial SD of bathymetry.

Models were cross-validated by space and time, and predictive performance was evaluated on an independent dataset consisting of more than 1 million records collated across public, private, and government sources (*17*). Model predictions were spatially constrained within a minimum bounding polygon around the telemetry data used to fit the models to avoid spatial extrapolation. Habitat suitability predictions are calculated at 0.25° and range from 0 (least suitable habitat) to 1 (most suitable habitat).

#### AIS data

##### Observed fishing vessel activity

AIS messages were acquired from Global Fishing Watch (GFW) from 2017 to 2022. We only examined the time series beginning in 2017 because this portion of the GFW AIS database contains data from both Orbcomm and Spire AIS providers, providing more complete coverage of fishing vessel activity. GFW uses two neural networks to identify fishing versus nonfishing behaviors and to identify the gear types used by fishing vessels (*6*). The final observed fishing vessel activity dataset included hours of fishing vessel activity (including vessels actively fishing and nonfishing behaviors such as transiting) by day, latitude, longitude, flag state, and gear type at a resolution of 0.25°.

##### AIS gap events

AIS gaps events are periods of missing data due to technical issues such as poor satellite coverage and low device ping rate or vessels intentionally disabling their AIS devices. Only periods that lasted longer than 12 hours were considered AIS gap events, with all other vessel activity deemed observable on AIS because satellite reception in a given area is relatively constant above this threshold [for full details, see (*16*)]. The final AIS gaps dataset included, for each gap event, the starting and ending position/time stamp, flag state, gear type, and a logical TRUE/FALSE for whether the gap was an intentional disabling event or not (see the “Intentional disabling events” section).

##### Intentional disabling events

Intentional disabling events are a subset of gap events, in which vessels are believed to have intentionally disabled their AIS devices. To identify intentional disabling events, gap events in waters of extremely poor satellite reception quality (<10 positions/day) were removed (*16*). Gap events that started or ended in waters 50 nautical miles from shore were also removed to control for gaps due to the transition between terrestrial and satellite based receivers. Next, a rule-based classification algorithm was applied to the remaining gaps to identify events, in which we have the most confidence, that were caused by intentional disabling as a function of AIS device ping rate and satellite reception quality. A series of rule-based classification algorithms based on a range of thresholds for ping rate and reception quality were tested against a labeled test set of intentional disabling events from an independent AIS data provider—ExactEarth (*16*). The algorithm with the highest precision and lowest false-positive rate was selected and applied to the gaps dataset to identify intentional disabling events.

##### Unseen fishing vessel activity

We spatially allocated the time (hours) and dates between the start and end of gaps events using linear interpolation at 1° resolution. While linear transits are unlikely, almost all activity in gaps shorter than 1 week (84% of all gap events) is within 1° of a line between gap starting and ending position, and 80% of activity in gaps longer than 1 week and shorter than 2 weeks is within 1° of this line (*16*). The percentage of unseen fishing vessel activity within 1° of a line between starting and ending positions decreases as gap length increases above 2 weeks, and therefore, we only map gap activity under 2 weeks (Figs. 1, 3, and 4) but calculate statistics for both gaps under 2 weeks (lower bound) and all gaps regardless of length (upper bound). The final unseen fishing vessel activity dataset included hours of activity by day, latitude, longitude, flag state, gear type, disabling event status (TRUE/FALSE), and gap length (over/under 2 weeks) at a resolution of 1°.

### Overlap between species distribution and fishing vessel activity

#### Species distribution data extraction

Observed and unseen fishing vessel activity datasets were clipped to the northeast Pacific (10°N to 60°N, −180°E to −100°E) to match the prediction domain of the species datasets. Habitat suitability predictions for each species were space/time-matched and extracted to the observed and unseen fishing vessel activity datasets. Habitat suitability was space/time-matched to the latitudes and longitudes in the observed fishing vessel activity dataset, as both datasets were at 0.25°. For unseen fishing vessel activity, which was at a resolution of 1°, habitat suitability was matched by averaging predictions within a 1° window around the latitudes and longitudes in the dataset. Next, observed fishing activity was aggregated to the same 1° grid as unseen fishing vessel activity. The two datasets were joined to produce a final dataset that included day, latitude, longitude, observed fishing vessel activity, unseen fishing vessel activity, disabling event status (TRUE/FALSE), gap length (over/under 2 weeks), flag state, gear type, and habitat suitability for each species.

#### Overlap calculations

Values for observed and unseen fishing vessel activity were rescaled between 0 and 1 as a single vector to preserve the differences in magnitude between the two datasets. Rescaling between 0 and 1 was done to match the range of species habitat suitability. Then, several different subsets of the rescaled observed and unseen fishing activity data were multiplied by habitat suitability for each species to produce:

1) Observed overlap: Observed fishing vessel activity × habitat suitability.

2) Unseen overlap: Unseen fishing vessel activity × habitat suitability.

3) Unseen overlap with disabling activity: Unseen fishing activity where disabling is TRUE × habitat suitability.

Observed overlap (1) captures the overlap between fishing vessel activity and species that is observable on AIS; unseen overlap (2) captures the overlap between fishing vessel activity and species that is obscured by gaps (both technical and intentional) in AIS data; unseen overlap with disabling activity (3) captures the overlap between fishing vessel activity and species that is obscured by intentional AIS disabling. Overlap metrics informed by unseen activity (2 and 3) included both lower and upper estimation bounds, in which gaps over 2 weeks in length are excluded and included, respectively, to account for uncertainty in spatial allocation (see the “Intentional disabling events” section)

Several additional derived metrics were calculated, including:

4) Estimated total overlap: Observed overlap (1) + unseen overlap (2).

5) Percent of overlap obscured by unseen fishing vessel activity: Unseen overlap (2)/estimated total overlap (4).

6) Percent of overlap obscured by disabling: Unseen overlap with disabling activity (3)/estimated total overlap (4).

Estimated total overlap (4) captures total overlap between fishing vessel activity and species when both observed and unseen overlap are accounted for. Percent of overlap obscured by unseen fishing vessel activity (5) captures the proportion of estimated total overlap that is obscured by unseen activity. Percent of overlap obscured by disabling (6) captures the proportion of estimated total overlap that is obscured by intentional AIS disabling. As above, metrics 4 to 6 include both lower and upper estimation bounds to account for spatial allocation uncertainty.

### Figure analyses

#### Figure 1

Bivariate mapping was used to display both estimated total overlap (*y* axis) summed across all species and the percent of overlap obscured by unseen fishing vessel activity (*x* axis). The lower bound (gaps over 2 weeks in length excluded) was used for both metrics to control for spatial allocation uncertainty. The *y* axis controls transparency, with higher and lower estimated total overlap equating to lower and higher transparency respectively, while the *x* axis controls color, with higher and lower percent of overlap obscured equating to yellow and blue, respectively. For ease of interpretation, estimated total overlap (*y* axis) was expressed simply as “higher” and “lower” because overlap is unitless. Version 11 of EEZ boundaries from marineecoregions.org (*67*) is displayed.

#### Figure 2

Panel (A) shows the percent of each species’ total habitat coinciding with observed fishing vessel activity, and the lower and upper estimation bounds of unseen fishing vessel activity. For each of 14 species, four calculations were done (Eqs. 1 to 4). Total habitat *TH* was calculated as the sum of suitability *S* across areas *a* and times *t*

$$\text{TH}=\sum_{a}\sum_{t}S_{a,t}$$ (1)

The percent of *TH* coinciding with observed fishing vessel activity %*H_obs_* was calculated as the sum of *S* at areas *a_obs* and times *t_obs*, where observed fishing vessel activity occurred and divided by *TH* and multiplied by 100

$$\%H_{\mathrm{obs}}=\frac{\sum_{a}\sum_{t}S_{a\_\mathrm{obs},t\_\mathrm{obs}}}{\mathrm{TH}}\times 100$$ (2)

The upper bound of the percent of *TH* coinciding with unseen fishing activity %*H_uu_* was calculated as the sum of *S* at areas *a_uu* and times *t_uu*, where unseen fishing vessel activity from gaps of all lengths occurred and divided by *TH* and multiplied by 100

$$\%H_{\mathrm{uu}}=\frac{\sum_{a}\sum_{t}S_{a\_\mathrm{uu},t\_\mathrm{uu}}}{\mathrm{TH}}\times 100$$ (3)

The lower bound of the percent of *TH* coinciding with unseen fishing activity %*H_ul_* was calculated as the sum of *S* at areas *a_ul* and times *t_ul*, where unseen fishing vessel activity from gaps under 2 weeks occurred and divided by *TH* and multiplied by 100

$$\%H_{\mathrm{ul}}=\frac{\sum_{a}\sum_{t}S_{a\_\mathrm{ul},t\_\mathrm{ul}}}{\mathrm{TH}}\times 100$$ (4)

Panel (B) shows the intensity in hours of observed fishing vessel activity and the lower and upper estimation bounds of unseen fishing vessel activity in each species’ habitat. For each of 14 species, three calculations were done (Eqs. 5 to 7).

Intensity of observed fishing vessel activity coinciding with species habitat *I_obs_* was calculated as the sum of observed hours *T_obs* at areas *a_suit* and times *t_suit*, where *S* > 0

$$I_{\mathrm{obs}}=\sum_{a}\sum_{t}T\_{\mathrm{obs}}_{a\_\text{suit},t\_\text{suit}}$$ (5)

The upper bound intensity of unseen fishing vessel activity coinciding with species habitat *I_uu_* was calculated as the sum of unseen hours from gaps of all lengths *T_uu* at areas *a_suit* and times *t_suit*, where *S* > 0

$$I_{\mathrm{uu}}=\sum_{a}\sum_{t}T\_{\mathrm{uu}}_{a\_\text{suit},t\_\text{suit}}$$ (6)

The lower bound intensity of unseen fishing vessel activity coinciding with species habitat *I_ul_* was calculated as the sum of unseen hours from gaps under 2 weeks *T_ul* at areas *a_suit* and times *t_suit* where *S* > 0

$$I_{\mathrm{ul}}=\sum_{a}\sum_{t}T\_{\mathrm{ul}}_{a\_\text{suit},t\_su\mathrm{it}}$$ (7)

Panel (C) shows the percent of estimated total overlap obscured by unseen overlap for each species. The lower estimation bound (gaps under 2 weeks) and upper estimation bound (all gap lengths) are shown for each species.

#### Figure 3

The extracted dataset (see the “Species distribution data extraction” section) was filtered to leatherback turtles and Laysan albatross. As in Fig. 1, bivariate mapping was used to display both estimated total overlap (*y* axis) and the percent of estimated total overlap obscured by unseen fishing activity (*x* axis; panels A and B). The *x* axis is directly comparable across maps, while the *y* axis has been scaled individually for each species to account for differences in overlap magnitudes (Laysan albatross have lower estimated total overlap than leatherbacks).

For panels (C) and (D), unseen overlap for each species was summarized by flag state and geographic location (EEZs and the high seas). For each species, chord diagrams were used to show the relative amounts of unseen overlap by flag state (yellow arc size), the relative amounts of unseen overlap by geographic location (blue arc size), and the relative amounts of unseen overlap in each geographic location by each flag state (arrow size).

#### Figure 4

IATTC data on shark catch in longline and tuna purse seine fleets were downloaded from www.iattc.org/en-US/Data/Public-domain. The dataset was restricted to mako and blue sharks to match the top predators in this present study—there were no reports of salmon or white sharks in our study domain. Longline data were available at 5° resolution from 2017 to 2021 and contained 813,325 mako and blue sharks; tuna purse seine data were available at 1° resolution from 2017 to 2022 and contained 2187 mako and blue sharks. The tuna purse seine data were aggregated to 5° and restricted to 2017 to 2021 to match the longline data. Shark data from the Western & Central Pacific Fisheries Commission was not used because it is not reported by flag state.

To focus the analysis on potential reporting discrepancies, U.S.-flagged vessels were removed from both datasets. AIS is required on U.S. vessels over 65 feet in length, operating in U.S. navigable waters (<12 nm from shore) (*68*). U.S.-flagged vessels have strict VMS requirements and high observer coverage, and thus, U.S. fishing activity that is unseen in the AIS record is likely observable from alternative data sources such as VMS. Second, previous work found that intentional AIS disabling by U.S.-flagged vessels in the northeast Pacific is primarily done to obscure the locations of high-quality fishing grounds, as opposed to obscuring nefarious activities (*16*). Last, the U.S. longline fleet is the only longline fleet to report its full shark catch rates to the IATTC (*25*).

The extracted dataset (see the “Species distribution data extraction” section) was filtered to mako and blue sharks and restricted to locations in time that contained unseen overlap with the lower estimation bound of disabling activity (gaps longer than 2 weeks excluded). Then, data were restricted to 2017 to 2021 and aggregated to 5° to match the IATTC data. The spatial domains of both the IATTC data and unseen overlap data were restricted to their area of intersection (10°N to 60°N, −150°E to −100°E). Both datasets are presented in map form in panels (A) and (B).

Panel (C) shows a scatter plot of the IATTC data versus the unseen overlap data, with a line of best fit and Pearson’s correlation coefficient calculated across the full datasets of each. To control for the effect of areas in which reporting discrepancies may be occurring on the expected relationship between unseen overlap and IATTC data, a second line of best fit and correlation coefficient was calculated after removing seven 5° grid cells with high unseen overlap and low IATTC catch. This allowed us to estimate a range of potential reporting discrepancies.

## Supplementary Materials

### This PDF file includes:

Figs. S1 to S8 Table S1 References
