# Supplementary material for: Unseen overlap between fishing vessels and top predators in the northeast Pacific
Source: Sci Adv. 2024 Mar 6;10(10):eadl5528. doi: 10.1126/sciadv.adl5528 (PMC10917345; doi:10.1126/sciadv.adl5528)
Supplement: Supplementary file 1 — Figs. S1 to S8 Table S1 References [file sciadv.adl5528_sm.pdf]

Science Advances
AAAS

# Supplementary Materials for

## Unseen overlap between fishing vessels and top predators in the northeast Pacific

Heather Welch *et al.*

Corresponding author: Heather Welch, heather.welch@noaa.gov

*Sci. Adv.* **10**, eadl5528 (2024)
DOI: 10.1126/sciadv.adl5528

**This PDF file includes:**

Figs. S1 to S8
Table S1
References

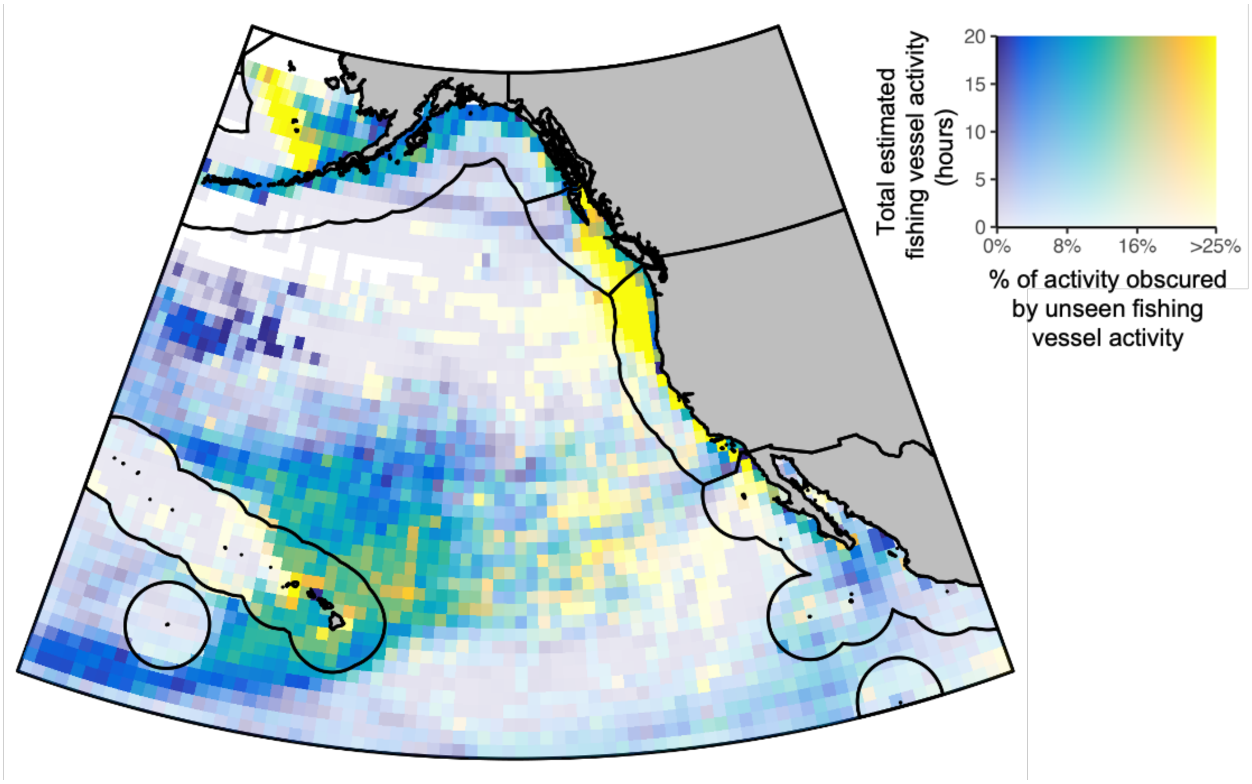


***Fig. S1. Estimated total fishing vessel activity and the percentage of this activity obscured by unseen fishing vessel activity***. *Black lines indicate Exclusive Economic Zone boundaries. Top predators include target species: yellowfin, albacore, and bluefin tunas; non-target species: blue, mako, and salmon sharks; and bycatch species: blue whales, elephant seals, California sea-lions, leatherback turtles, white sharks, Laysan and black-footed albatrosses, and sooty shearwaters.*

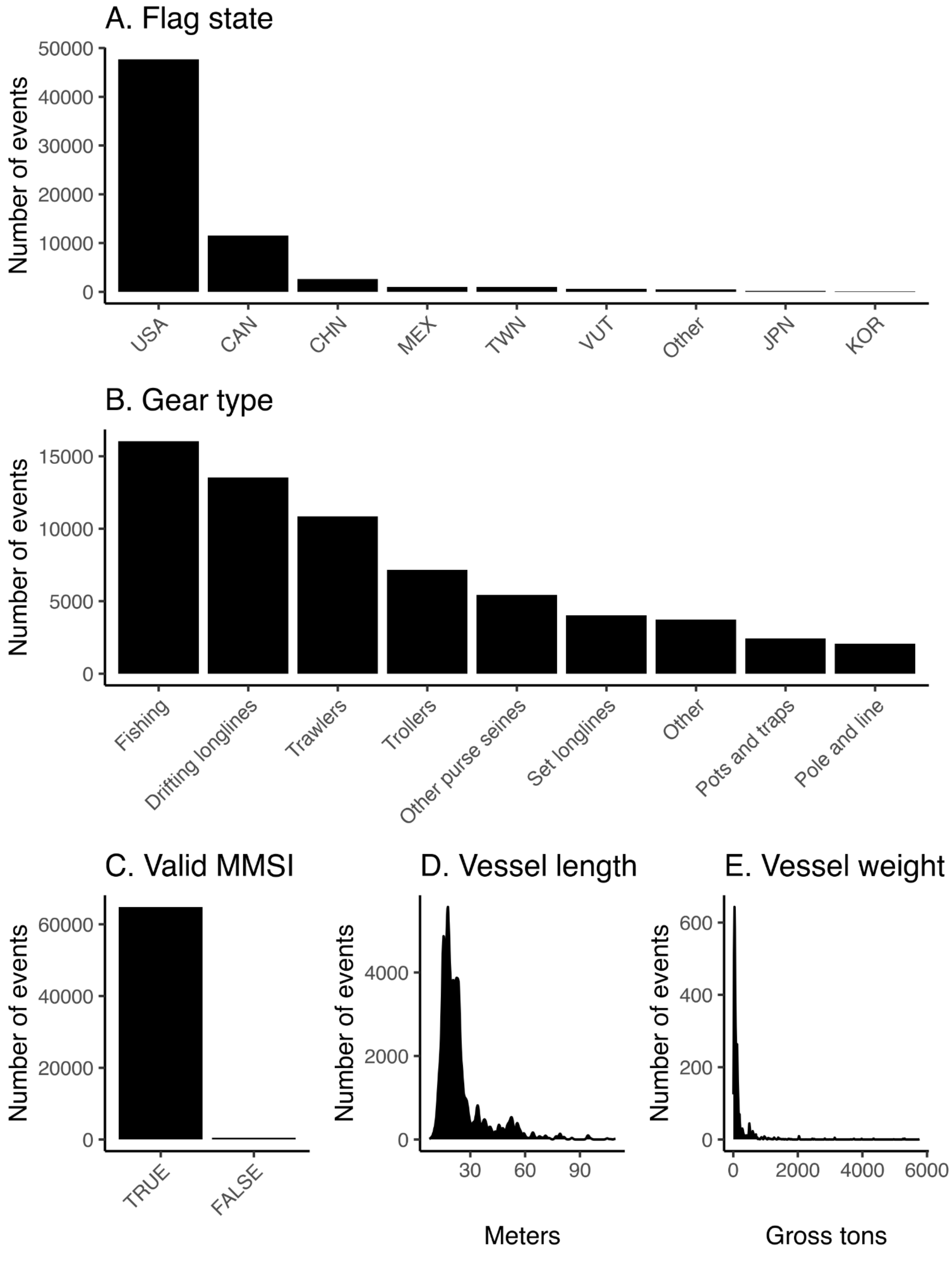


***Fig. S2. Gap events by the numbers.*** *A) Number of events by flag state: Canada (CAN), China (CHN), Mexico (Mex), Chinese Taipei (TWN), Vanuatu (VUT), Japan (JPN), South Korea (KOR). B) Number of events by gear type: Fishing indicates that the specific gear type could not be identified. C) Number of events with valid Maritime Mobile Service Identity (MMSI) numbers: TRUE/FALSE based on if the maritime identification digits (the first three digits of the MMSI number) are valid. D) Histograms of vessel length in meters and E) vessel weight in gross tons.*

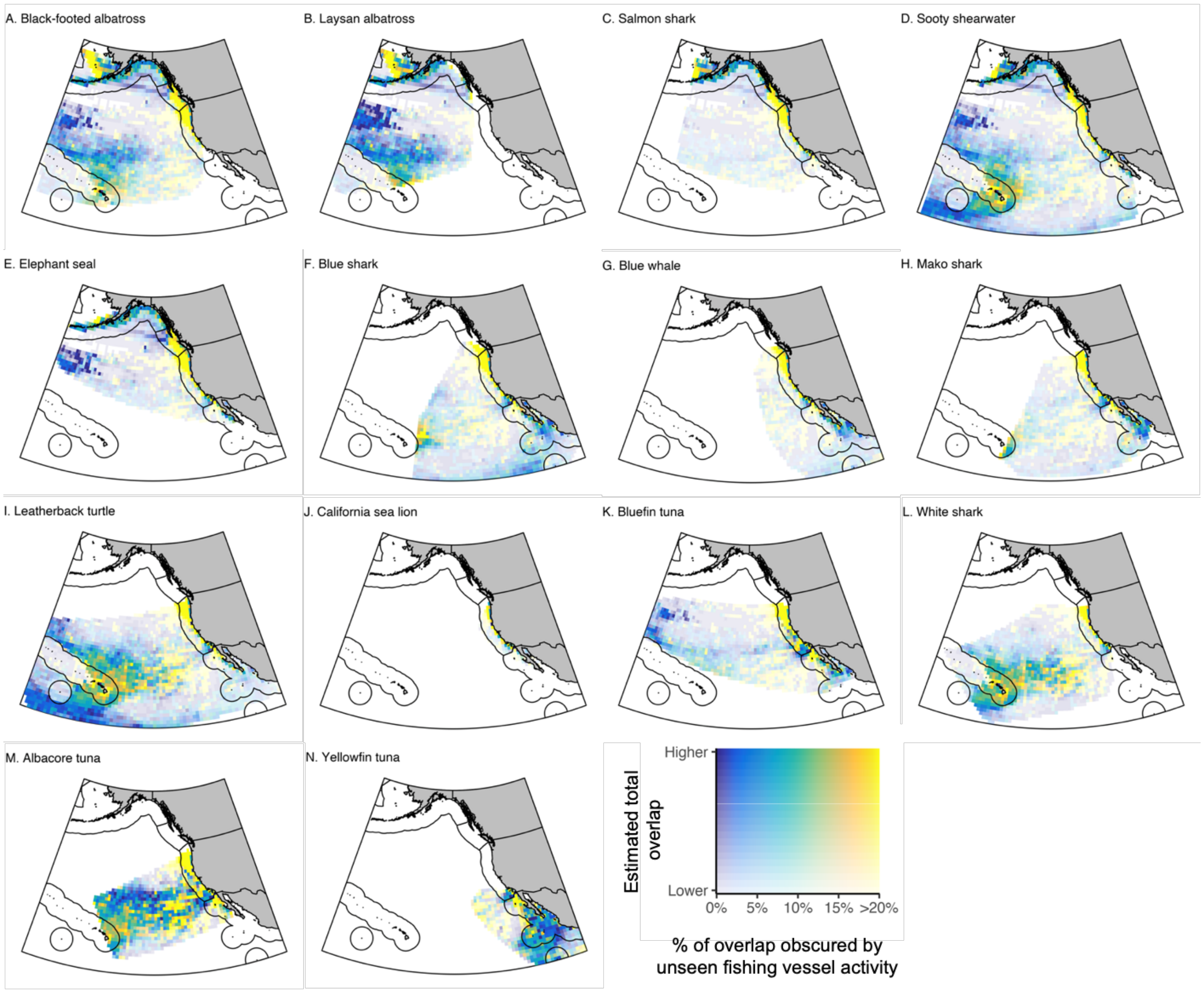


***Fig. S3. Estimated total overlap and the percentage of this overlap obscured by unseen fishing vessel activity for each species.*** *Black lines indicate Exclusive Economic Zone boundaries. Top predators black-footed albatross (A), Laysan albatross (B), salmon shark (C), sooty shearwater (D), elephant seal (E), blue shark (F), blue whale (G), mako shark (H), leatherback turtle (I), California sea lion (J), bluefin tuna (K), white shark (L), albacore tuna (M), yellowfin tuna (N).*

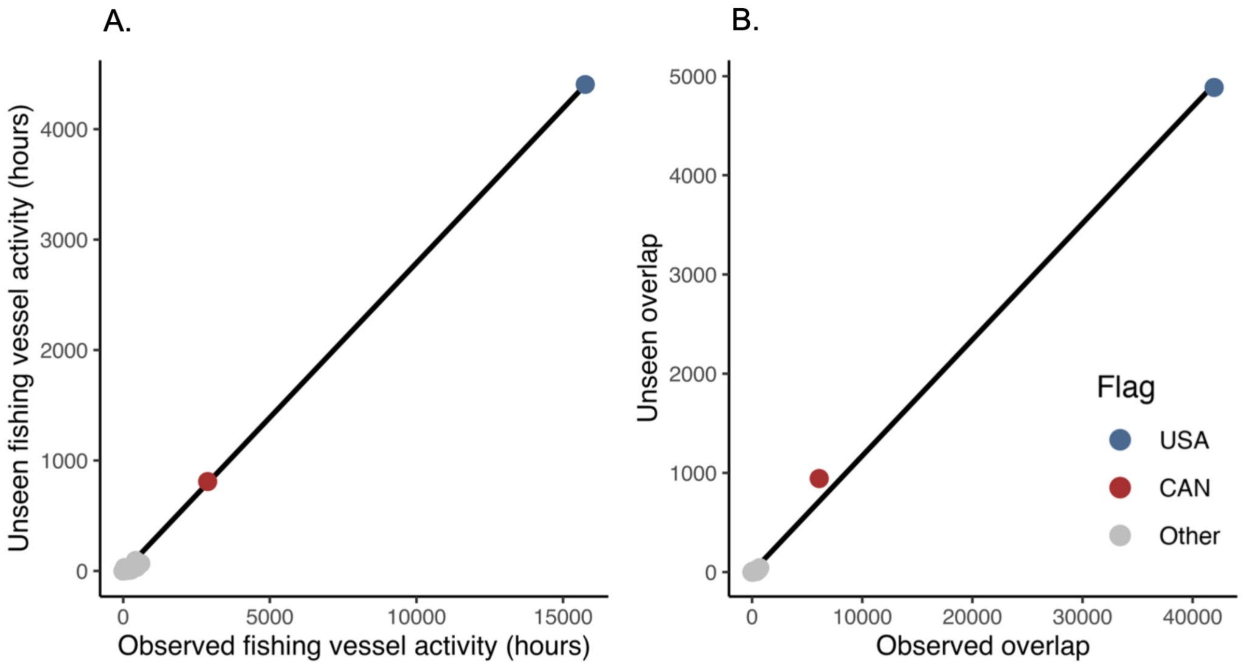


***Fig. S4. Observed and unseen fishing vessel activity and overlap by flag state.*** *A) Observed fishing vessel activity versus unseen fishing vessel activity; B) observed overlap versus unseen overlap. Black line in both A) and B) is line of best fit; unseen fishing vessel activity and unseen overlap are both calculated using upper estimation bounds, i.e. gaps longer than two weeks included; Canada (CAN).*

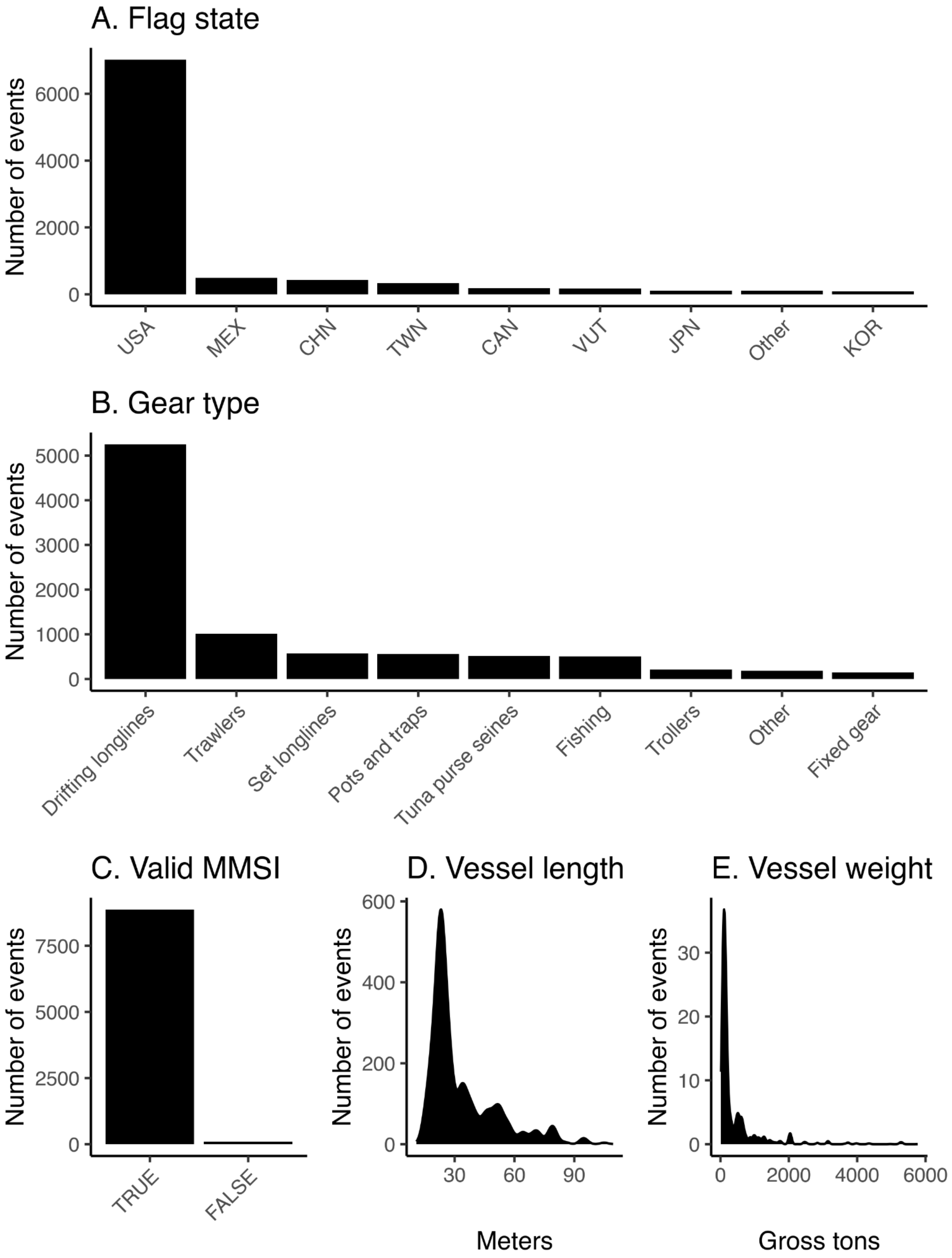


***Fig. S5. Intentional disabling events by the numbers.*** *A) Number of events by flag state: Mexico (MEX), China (CHN), Chinese Taipei (TWN), Canada (CAN), Vanuatu (VUT), Japan (JPN), South Korea (KOR). B) Number of events by gear type: Fishing indicates that the specific gear type could not be identified. C) Number of events with valid Maritime Mobile Service Identity (MMSI) numbers: TRUE/FALSE based on if the maritime identification digits (the first three digits of the MMSI number) are valid. D) Histograms of vessel length in meters and E) vessel weight in gross tons.*

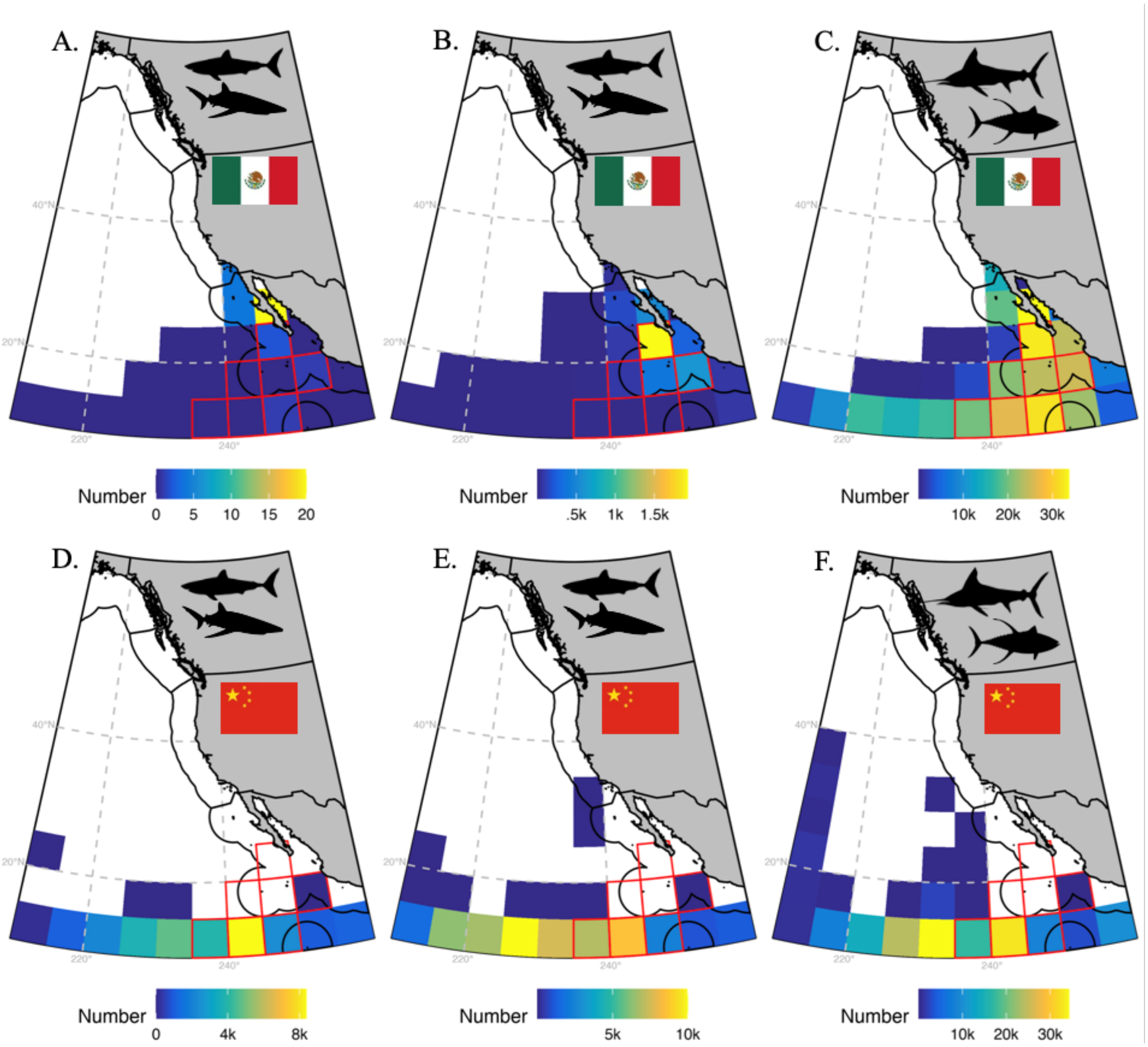


***Fig. S6. Longline and purse seine catch reported to the Inter-American Tropical Tuna Commission.*** *Mako and blue shark catch reported by Mexico (A.) and China (D.) from 2017-2021. All available reported mako and blue shark catch from Mexico (1981-2022; B.) and China (2010-2021; E.). Tuna and billfish catch reported by Mexico (C.) and China (F.) from 2017-2021. Areas outlined in red match those in Figure 4. Catch data were downloaded from https://www.iattc.org/en-US/Data/Public-domain.*

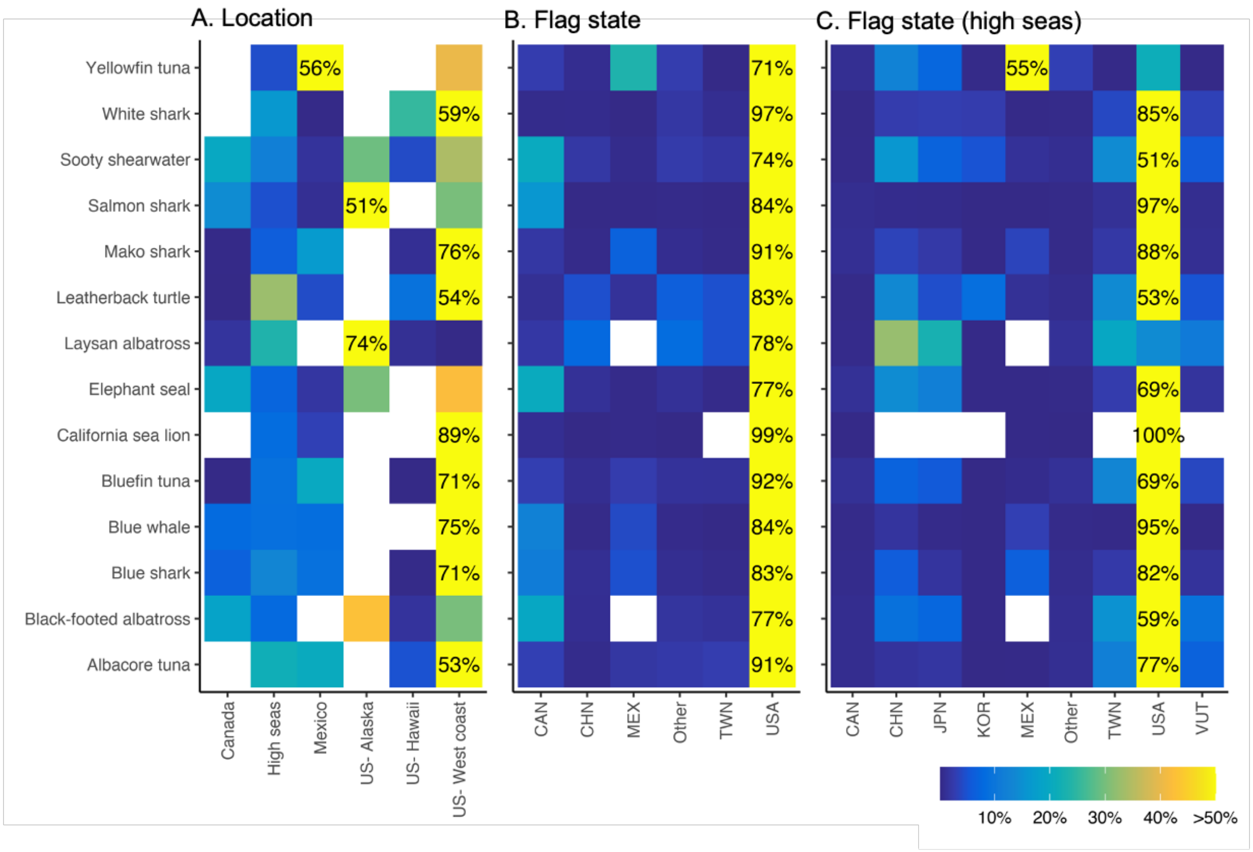


***Fig. S7. Observed overlap between top predators and fishing vessels.*** *For each species, the proportion of observed overlap in the northeast Pacific by geopolitical location (A.) and flag state (B.), e.g. 56% and 71% of observed yellowfin tuna overlap occurred in Mexican waters and with US flagged vessels, respectively. For each species, panel C. shows the proportion of observed overlap in the high seas by flag state, e.g. 55% of observed yellowfin tuna overlap in the high seas occurred with Mexican flagged vessels. Percentages in each panel/row sum to 100. Country codes: Canada (CAN), China (CHN), Mexico (MEX), Chinese Taipei (TWN), Japan (JPN), South Korea (KOR), Vanuatu (VUT).*

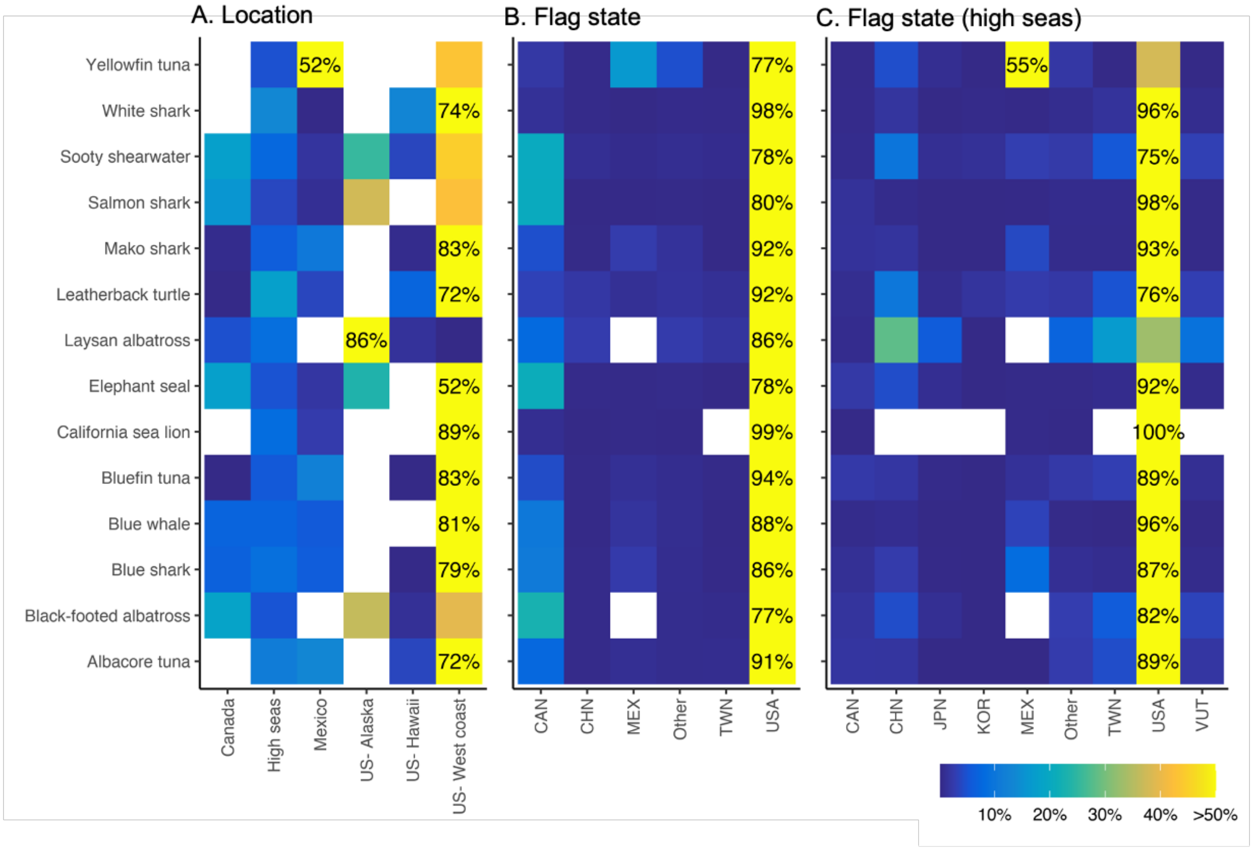


***Fig. S8. Unseen overlap between top predators and fishing vessels.*** *For each species, the proportion of unseen overlap in the northeast Pacific by geopolitical location (A.) and flag state (B.), e.g. 52% and 77% of unseen yellowfin tuna overlap occurred in Mexican waters and with US flagged vessels, respectively. For each species, panel C. shows the proportion of unseen overlap in the high seas by flag state, e.g. 55% of unseen yellowfin tuna overlap in the high seas occurred with Mexican flagged vessels. Percentages in each panel/row sum to 100. Country codes: Canada (CAN), China (CHN), Mexico (MEX), Chinese Taipei (TWN), Japan (JPN), South Korea (KOR), Vanuatu (VUT).*

*Table S1. Population statuses of the 14 top predators across different regulatory bodies. Under the Convention on International Trade in Endangered Species of Wild Fauna and Flora (CITES), Appendix I species are those currently threatened with extinction, and Appendix II species are those likely to become threatened with extinction if trade is not closely controlled. Under the International Union for the Conservation of Nature (IUCN), species are classified by population trend (decreasing, stable, increasing) and conservation status: least concern (LC), near threatened (NT), vulnerable (VU), endangered (EN), and critically endangered (CR). Species listed in Appendix I of the Convention on the Conservation of Migratory Species of Wild Animals (CMS) are those that are currently threatened with extinction, and species listed in Appendix II are those that need or would greatly benefit from international co-operation. Under the Endangered Species Act (ESA), both blue whales and leatherback turtles are listed as endangered (EN). Where available, the stock status of the Northeast Pacific populations of each predator is presented along with the assessing agency: the International Seafood Sustainability Foundation (ISSF), the National Oceanic and Atmospheric Administration (NOAA), the International Scientific Committee for Tuna and Tuna-like Species in the North Pacific Ocean (ISC), and the United States Geological Survey (USGS).*

| | **Species** | **Sci name** | **CITES** | **IUCN** | **CMS** | **ESA** | **Northeast Pacific stock status** |
|---|---|---|---|---|---|---|---|
| **Tunas** | Albacore tuna | *Thunnus alalunga* | | LC; Decreasing | | | Not overfishing or subject to overfishing. ISSF. (69) |
| | Bluefin tuna | *Thunnus orientalis* | | NT; Decreasing | | | Heavily overfished. ISSF. (69) |
| | Yellofin tuna | *Thunnus albacares* | | LC; Decreasing | | | Not overfishing or subject to overfishing. ISSF. (69) |
| **Mammals** | Blue whale | *Balaenoptera musculus* | Appendix I | EN; Increasing | Appendix I | EN | Depleated. NOAA. (70) |
| | California sea lion | *Zalophus californianus* | | LC; Increasing | | | Not depleated. NOAA. (70) |
| | Elephant seal | *Mirounga angustirostris* | Appendix II | LC; Increasing | | | Not depleated. NOAA. (70) |
| **Sharks** | Blue shark | *Prionace glauca* | | NT; Decreasing | Appendix II | | Not overfishing or subject to overfishing. ISC. (71) |
| | Mako shark | *Isurus oxyrinchus* | Appendix II | EN; Increasing | Appendix II | | Not overfishing or subject to overfishing. ISC. (72) |
| | Salmon shark | *Lamna ditropis* | | LC; Stable | | | N/A |
| | White shark | *Carcharodon carcharias* | Appendix II | VU; Decreasing | Appendix I | | Stable or increasing. NOAA. (73) |
| **Seabirds** | Black-footed albatross | *Phoebastria nigripes* | | NT; Increasing | Appendix II | | Stable. USGS. (74) |
| | Laysan albatross | *Phoebastria immutabilis* | | NT; Stable | Appendix II | | Increasing. USGS. (74) |
| | Sooty shearwater | *Ardenna grisea* | | NT; Decreasing | | | N/A |
| **Sea turtle** | Leatherback turtle | *Dermochelys coriacea* | Appendix I | CR; Decreasing | Appendix I | EN | High extinction risk. NOAA. (75) |

## REFERENCES AND NOTES

1. R. L. Lewison, L. B. Crowder, B. P. Wallace, J. E. Moore, T. Cox, R. Zydelis, S. McDonald, A. DiMatteo, D. C. Dunn, C. Y. Kot, R. Bjorkland, S. Kelez, C. Soykan, K. R. Stewart, M. Sims, A. Boustany, A. J. Read, P. Halpin, W. J. Nichols, C. Safina, Global patterns of marine mammal, seabird, and sea turtle bycatch reveal taxa-specific and cumulative megafauna hotspots. *Proc. Natl. Acad. Sci.* **111**, 5271–5276 (2014).

2. R. L. Lewison, L. B. Crowder, A. J. Read, S. A. Freeman, Understanding impacts of fisheries bycatch on marine megafauna. *Trends Ecol. Evol.* **19**, 598–604 (2004).

3. B. B. Collette, K. E. Carpenter, B. A. Polidoro, M. J. Juan-Jordá, A. Boustany, D. J. Die, C. Elfes, W. Fox, J. Graves, L. R. Harrison, R. McManus, C. V. Minte-Vera, R. Nelson, V. Restrepo, J. Schratwieser, C.-L. Sun, A. Amorim, M. Brick Peres, C. Canales, G. Cardenas, S.-K. Chang, W.-C. Chiang, N. de Oliveira Leite, H. Harwell, R. Lessa, F. L. Fredou, H. A. Oxenford, R. Serra, K.-T. Shao, R. Sumaila, S.-P. Wang, R. Watson, E. Yáñez, High Value and Long Life—Double Jeopardy for Tunas and Billfishes. *Science* **333**, 291–292 (2011).

4. J. A. Santora, N. J. Mantua, I. D. Schroeder, J. C. Field, E. L. Hazen, S. J. Bograd, W. J. Sydeman, B. K. Wells, J. Calambokidis, L. Saez, D. Lawson, K. A. Forney, Habitat compression and ecosystem shifts as potential links between marine heatwave and record whale entanglements. *Nat. Commun.* **11**, 536 (2020).

5. S. D. Kraus, M. W. Brown, H. Caswell, C. W. Clark, M. Fujiwara, P. K. Hamilton, R. D. Kenney, A. R. Knowlton, S. Landry, C. A. Mayo, W. A. McLellan, M. J. Moore, D. P. Nowacek, D. A. Pabst, A. J. Read, R. M. Rolland, North Atlantic Right Whales in Crisis. *Science* **309**, 561–562 (2005).

6. D. A. Kroodsma, J. Mayorga, T. Hochberg, N. A. Miller, K. Boerder, F. Ferretti, A. Wilson, B. Bergman, T. D. White, B. A. Block, P. Woods, B. Sullivan, C. Costello, B. Worm, Tracking the global footprint of fisheries. *Science* **359**, 904–908 (2018).

7. G. O. Crespo, D. C. Dunn, G. Reygondeau, K. Boerder, B. Worm, W. Cheung, D. P. Tittensor, P. N. Halpin, The environmental niche of the global high seas pelagic longline fleet. *Sci. Adv.* **4**, eaat3681 (2018).

8. T. D. White, F. Ferretti, D. A. Kroodsma, E. L. Hazen, A. B. Carlisle, K. L. Scales, S. J. Bograd, B. A. Block, Predicted hotspots of overlap between highly migratory fishes and industrial fishing fleets in the northeast Pacific. *Sci. Adv.* **5**, eaau3761 (2019).

9. H. Blondin, B. Abrahms, L. B. Crowder, E. L. Hazen, Combining high temporal resolution whale distribution and vessel tracking data improves estimates of ship strike risk. *Biol. Conserv.* **250**, 108757 (2020).

10. D. Bradley, J. Mayorga, D. J. McCauley, R. B. Cabral, P. Douglas, S. D. Gaines, Leveraging satellite technology to create true shark sanctuaries. *Conserv. Lett.* **12**, e12610 (2019).

11. R. A. Orben, J. Adams, M. Hester, S. A. Shaffer, R. M. Suryan, T. Deguchi, K. Ozaki, F. Sato, L. C. Young, C. Clatterbuck, M. G. Conners, D. A. Kroodsma, L. G. Torres, Across borders: External factors and prior behaviour influence North Pacific albatross associations with fishing vessels. *J. Appl. Ecol.* **58**, 1272–1283 (2021).

12. A. P. B. Carneiro, B. L. Clark, E. J. Pearmain, T. Clavelle, A. G. Wood, R. A. Phillips, Finescale associations between wandering albatrosses and fisheries in the southwest Atlantic Ocean. *Biol. Conserv.* **276**, 109796 (2022).

13. F. C. Womersley, N. E. Humphries, N. Queiroz, M. Vedor, I. Da Costa, M. Furtado, J. P. Tyminski, K. Abrantes, G. Araujo, S. S. Bach, A. Barnett, M. L. Berumen, S. Bessudo Lion, C.

D. Braun, E. Clingham, J. E. M. Cochran, R. de la Parra, S. Diamant, A. D. M. Dove, C. L. Dudgeon, M. V. Erdmann, E. Espinoza, R. Fitzpatrick, J. G. Cano, J. R. Green, H. M. Guzman, R. Hardenstine, A. Hasan, F. H. V. Hazin, A. R. Hearn, R. E. Hueter, M. Y. Jaidah, J. Labaja, F. Ladino, B. C. L. Macena, J. J. Morris, B. M. Norman, C. Peñaherrera-Palma, S. J. Pierce, L. M. Quintero, D. Ramírez-Macías, S. D. Reynolds, A. J. Richardson, D. P. Robinson, C. A. Rohner, D. R. L. Rowat, M. Sheaves, M. S. Shivji, A. B. Sianipar, G. B. Skomal, G. Soler, I. Syakurachman, S. R. Thorrold, D. H. Webb, B. M. Wetherbee, T. D. White, T. Clavelle, D. A. Kroodsma, M. Thums, L. C. Ferreira, M. G. Meekan, L. M. Arrowsmith, E. K. Lester, M. M. Meyers, L. R. Peel, A. M. M. Sequeira, V. M. Eguíluz, C. M. Duarte, D. W. Sims, Global collision-risk hotspots of marine traffic and the world's largest fish, the whale shark. *Proc. Natl. Acad. Sci.* **119**, e2117440119 (2022).

14. N. Queiroz, N. E. Humphries, A. Couto, M. Vedor, I. Da Costa, A. M. M. Sequeira, G. Mucientes, A. M. Santos, F. J. Abascal, D. L. Abercrombie, K. Abrantes, D. Acuña-Marrero, A. S. Afonso, P. Afonso, D. Anders, G. Araujo, R. Arauz, P. Bach, A. Barnett, D. Bernal, M. L. Berumen, S. Bessudo Lion, N. P. A. Bezerra, A. V. Blaison, B. A. Block, M. E. Bond, R. Bonfil, R. W. Bradford, C. D. Braun, E. J. Brooks, A. Brooks, J. Brown, B. D. Bruce, M. E. Byrne, S. E. Campana, A. B. Carlisle, D. D. Chapman, T. K. Chapple, J. Chisholm, C. R. Clarke, E. G. Clua, J. E. M. Cochran, E. C. Crochelet, L. Dagorn, R. Daly, D. D. Cortés, T. K. Doyle, M. Drew, C. A. J. Duffy, T. Erikson, E. Espinoza, L. C. Ferreira, F. Ferretti, J. D. Filmalter, G. C. Fischer, R. Fitzpatrick, J. Fontes, F. Forget, M. Fowler, M. P. Francis, A. J. Gallagher, E. Gennari, S. D. Goldsworthy, M. J. Gollock, J. R. Green, J. A. Gustafson, T. L. Guttridge, H. M. Guzman, N. Hammerschlag, L. Harman, F. H. V. Hazin, M. Heard, A. R. Hearn, J. C. Holdsworth, B. J. Holmes, L. A. Howey, M. Hoyos, R. E. Hueter, N. E. Hussey, C. Huveneers, D. T. Irion, D. M.

P. Jacoby, O. J. D. Jewell, R. Johnson, L. K. B. Jordan, S. J. Jorgensen, W. Joyce, C. A. Keating Daly, J. T. Ketchum, A. P. Klimley, A. A. Kock, P. Koen, F. Ladino, F. O. Lana, J. S. E. Lea, F. Llewellyn, W. S. Lyon, A. MacDonnell, B. C. L. Macena, H. Marshall, J. D. McAllister, R. McAuley, M. A. Meÿer, J. J. Morris, E. R. Nelson, Y. P. Papastamatiou, T. A. Patterson, C. Peñaherrera-Palma, J. G. Pepperell, S. J. Pierce, F. Poisson, L. M. Quintero, A. J. Richardson, P. J. Rogers, C. A. Rohner, D. R. L. Rowat, M. Samoilys, J. M. Semmens, M. Sheaves, G. Shillinger, M. Shivji, S. Singh, G. B. Skomal, M. J. Smale, L. B. Snyders, G. Soler, M. Soria, K. M. Stehfest, J. D. Stevens, S. R. Thorrold, M. T. Tolotti, A. Towner, P. Travassos, J. P. Tyminski, F. Vandeperre, J. J. Vaudo, Y. Y. Watanabe, S. B. Weber, B. M. Wetherbee, T. D. White, S. Williams, P. M. Zárate, R. Harcourt, G. C. Hays, M. G. Meekan, M. Thums, X. Irigoien, V. M. Eguiluz, C. M. Duarte, L. L. Sousa, S. J. Simpson, E. J. Southall, D. W. Sims, Global spatial risk assessment of sharks under the footprint of fisheries. *Nature* **572**, 461–466 (2019).

15. R. C. Rockwood, J. Calambokidis, J. Jahncke, High mortality of blue, humpback and fin whales from modeling of vessel collisions on the U.S. West Coast suggests population impacts and insufficient protection. *PLOS ONE* **12**, e0183052 (2017).

16. H. Welch, T. Clavelle, T. D. White, M. A. Cimino, J. Van Osdel, T. Hochberg, D. Kroodsma, E. L. Hazen, Hot spots of unseen fishing vessels. *Sci. Adv.* **8**, eabq2109 (2022).

17. H. Welch, M. S. Savoca, S. Brodie, M. G. Jacox, B. A. Muhling, T. A. Clay, M. A. Cimino, S. R. Benson, B. A. Block, M. G. Conners, D. P. Costa, F. D. Jordan, A. W. Leising, C. S. Mikles, D. M. Palacios, S. A. Shaffer, L. H. Thorne, J. T. Watson, R. R. Holser, L. Dewitt, S. J. Bograd, E. L. Hazen, Impacts of marine heatwaves on top predator distributions are variable but predictable. *Nat. Commun.* **14**, 5188 (2023).

18. B. A. Block, I. D. Jonsen, S. J. Jorgensen, A. J. Winship, S. A. Shaffer, S. J. Bograd, E. L. Hazen, D. G. Foley, G. A. Breed, A.-L. Harrison, J. E. Ganong, A. Swithenbank, M. Castleton, H. Dewar, B. R. Mate, G. L. Shillinger, K. M. Schaefer, S. R. Benson, M. J. Weise, R. W. Henry, D. P. Costa, Tracking apex marine predator movements in a dynamic ocean. *Nature* **475**, 86–90 (2011).

19. A. J. Winship, S. J. Jorgensen, S. A. Shaffer, I. D. Jonsen, P. W. Robinson, D. P. Costa, B. A. Block, State-space framework for estimating measurement error from double-tagging telemetry experiments. *Methods Ecol. Evol.* **3**, 291–302 (2012).

20. F. D. Jordan, S. A. Shaffer, M. G. Conners, J. E. F. Stepanuk, M. E. Gilmour, C. A. Clatterbuck, E. L. Hazen, D. M. Palacios, Y. Tremblay, M. Antolos, D. G. Foley, S. J. Bograd, D. P. Costa, L. H. Thorne, Divergent post-breeding spatial habitat use of Laysan and blackfooted albatross. *Front. Ecol. Evol.* **10** (2022).

21. B. Abrahms, H. Welch, S. Brodie, M. G. Jacox, E. A. Becker, S. J. Bograd, L. M. Irvine, D. M. Palacios, B. R. Mate, E. L. Hazen, Dynamic ensemble models to predict distributions and anthropogenic risk exposure for highly mobile species. *Divers. Distrib.* **25**, 1182–1193 (2019).

22. E. L. Hazen, K. L. Scales, S. M. Maxwell, D. K. Briscoe, H. Welch, S. J. Bograd, H. Bailey, S. R. Benson, T. Eguchi, H. Dewar, S. Kohin, D. P. Costa, L. B. Crowder, R. L. Lewison, A dynamic ocean management tool to reduce bycatch and support sustainable fisheries. *Sci. Adv.* **4**, eaar3001 (2018).

23. M. S. Savoca, S. Brodie, H. Welch, A. Hoover, L. R. Benaka, S. J. Bograd, E. L. Hazen, Comprehensive bycatch assessment in US fisheries for prioritizing management. *Nat. Sustain.* **3**, 472–480 (2020).

24. A.-L. Harrison, D. P. Costa, A. J. Winship, S. R. Benson, S. J. Bograd, M. Antolos, A. B.

Carlisle, H. Dewar, P. H. Dutton, S. J. Jorgensen, S. Kohin, B. R. Mate, P. W. Robinson, K. M. Schaefer, S. A. Shaffer, G. L. Shillinger, S. E. Simmons, K. C. Weng, K. M. Gjerde, B. A. Block, The political biogeography of migratory marine predators. *Nat. Ecol. Evol.* **2**, 1571–1578 (2018).

25. S. Clarke, M. Sato, C. Small, B. Sullivan, D. Ochi, "Bycatch in Longline Fisheries for Tuna and Tuna- like Species: A Global Review of Status and Mitigation Measures" (Food and Agriculture Organization of the United Nations, WCPFC-SC10-2014/ EB-IP-04, 2014); www.bmis-bycatch.org/system/files/zotero_attachments/library_1/V8QFGZXH%20-%20EB-IP-04%20Global%20Review%20of%20LL%20Bycatch%20Mitigation.pdf. 26. J. V. Redfern, E. A. Becker, T. J. Moore, Effects of variability in ship traffic and whale distributions on the risk of ships striking whales. *Front. Mar. Sci.* **6**, 793 (2020).

27. D. W. Laist, A. R. Knowlton, J. G. Mead, A. S. Collet, M. Podesta, Collisions between ships and whales. *Mar. Mamm. Sci.* **17**, 35–75 (2001).

28. E. M. Keen, K. L. Scales, B. K. Rone, E. L. Hazen, E. A. Falcone, G. S. Schorr, Night and day: Diel differences in ship strike risk for fin whales (*Balaenoptera physalus*) in the California Current System. *Front. Mar. Sci.* **6**, 730 (2019).

29. J. T. Watson, R. Ames, B. Holycross, J. Suter, K. Somers, C. Kohler, B. Corrigan, Fishery catch records support machine learning-based prediction of illegal fishing off U.S. West Coast. *Peer J.* **11**, e16215 (2023)

30. J. M. Suter, R. T. Ames, B. Holycross, J. T. Watson, Comparing observed and unobserved fishing characteristics in the drift gillnet fishery for swordfish. *Fish. Res.* **256**, 106456 (2022).

31. S. Griffiths, C. E. Lennert-Cody, B. Wiley, L. Fuller, "Update on operational longline observer data required under resolution c-19-08 and a preliminary assessment of data reliability for estimating total

catch for bycatch species in the eastern pacific ocean," in 10th Meeting of the Working Group on Bycatch (2021), p. 25; www.iattc.org/getattachment/476948e6-a594-4bc7- a470-69303b6e14c2/BYC-10-INF-D_Update-on-operational-longline-observer-data.pdf.

32. WCPFC, "Conservation and management measure for the regional observer programme," in *15th Regular Session of the Western and Central Pacific Fisheries Commission* (2018); https://cmm.wcpfc.int/measure/cmm-2018-05.

33. J. Wang, X. Gao, J. Chen, X. Dai, S. Tian, Y. Chen, An evaluation of observer monitoring program designs for Chinese tuna longline fisheries in the Pacific Ocean using computer simulations. *Environ. Sci. Pollut. Res.* **28**, 12628–12639 (2021).

34. D. C. Dunn, C. Jablonicky, G. O. Crespo, D. J. McCauley, D. A. Kroodsma, K. Boerder, K. M. Gjerde, P. N. Halpin, Empowering high seas governance with satellite vessel tracking data. *Fish Fish.* **19**, 729–739 (2018).

35. K. N. Heidrich, M. J. Juan-Jordá, H. Murua, C. D. H. Thompson, J. J. Meeuwig, D. Zeller, Assessing progress in data reporting by tuna Regional Fisheries Management Organizations. *Fish Fish.* **23**, 1264–1281 (2022).

36. C. Pott, D. A. Wiedenfeld, Information gaps limit our understanding of seabird bycatch in global fisheries. *Biol. Conserv.* **210**, 192–204 (2017).

37. IATTC, "Resolution on data provision," in *70th meeting of Inter-American Tropical Tuna Commission* (2003); www.iattc.org/GetAttachment/743b7b81-fe91-41e1-9f67-670630408daf/C- 03-05-Active_Provision-of-data.pdf.

38. D. Pauly, D. Belhabib, R. Blomeyer, W. W. W. L. Cheung, A. M. Cisneros-Montemayor, D. Copeland, S. Harper, V. W. Y. Lam, Y. Mai, F. Le Manach, H. Österblom, K. M. Mok, L. van der Meer, A. Sanz, S. Shon, U. R. Sumaila, W. Swartz, R. Watson, Y. Zhai, D. Zeller, China's

distant-water fisheries in the 21st century. *Fish Fish.* **15**, 474–488 (2014).

39. S. Clarke, Use of shark fin trade data to estimate historic total shark removals in the Atlantic Ocean. *Aquat. Living Resour.* **21**, 373–381 (2008).

40. G. Mucientes, M. Vedor, D. W. Sims, N. Queiroz, Unreported discards of internationally protected pelagic sharks in a global fishing hotspot are potentially large. *Biol. Conserv.* **269**, 109534 (2022).

41. R. Watson, D. Pauly, Systematic distortions in world fisheries catch trends. *Nature* **414**, 534–536 (2001).

42. D. Kroodsma, J. Turner, C. Luck, T. Hochberg, N. Miller, P. Augustyn, S. Prince, Global prevalence of setting longlines at dawn highlights bycatch risk for threatened albatross. *Biol. Conserv.* **283**, 110026 (2023).

43. M. Taconet, D. A. Kroodsma, J. A. Fernandes, Global atlas of AIS-based fishing activity—Challenges and opportunities (2019).

44. F. Paolo, D. Kroodsma, J. Raynor, T. Hochberg, P. Davis, J. Cleary, L. Marsaglia, S. Orofino, C. Thomas, P. Halpin, Satellite mapping reveals extensive industrial activity at sea. *Nature* **625**, 85–91 (2024).

45. MSA, Magnuson-Stevens Fishery Conservation and Management Act. *Public Law* **94–265** (2007).

46. NOAA Fisheries, U.S. Fisheries Management: Sustainable Fisheries, Sustainable Seafood (2023); https://media.fisheries.noaa.gov/dam-migration/fisheries-management-msa-factsheet.pdf.

47. N. Queiroz, N. E. Humphries, G. Mucientes, N. Hammerschlag, F. P. Lima, K. L. Scales, P. I. Miller, L. L. Sousa, R. Seabra, D. W. Sims, Ocean-wide tracking of pelagic sharks reveals extent of overlap with longline fishing hotspots. *Proc. Natl. Acad. Sci.* **113**, 1582–1587 (2016).

48. S. M. Maxwell, E. L. Hazen, S. J. Bograd, B. S. Halpern, G. A. Breed, B. Nickel, N. M.

Teutschel, L. B. Crowder, S. Benson, P. H. Dutton, H. Bailey, M. A. Kappes, C. E. Kuhn, M. J. Weise, B. Mate, S. A. Shaffer, J. L. Hassrick, R. W. Henry, L. Irvine, B. I. McDonald, P. W. Robinson, B. A. Block, D. P. Costa, Cumulative human impacts on marine predators. *Nat. Commun.* **4**, 2688 (2013).

49. J. H. Roe, S. J. Morreale, F. V. Paladino, G. L. Shillinger, S. R. Benson, S. A. Eckert, H. Bailey, P. S. Tomillo, S. J. Bograd, T. Eguchi, P. H. Dutton, J. A. Seminoff, B. A. Block, J. R. Spotila, Predicting bycatch hotspots for endangered leatherback turtles on longlines in the Pacific Ocean. *Proc. R. Soc. B Biol. Sci.* **281**, 20132559 (2014).

50. R. J. Shucksmith, R. L. Shelmerdine, A risk based approach to non-native species management and biosecurity planning. *Mar. Policy* **59**, 32–43 (2015).

51. N. D. Merchant, M. J. Witt, P. Blondel, B. J. Godley, G. H. Smith, Assessing sound exposure from shipping in coastal waters using a single hydrophone and Automatic Identification System (AIS) data. *Mar. Pollut. Bull.* **64**, 1320–1329 (2012).

52. N. D. Merchant, E. Pirotta, T. R. Barton, P. M. Thompson, Monitoring ship noise to assess the impact of coastal developments on marine mammals. *Mar. Pollut. Bull.* **78**, 85–95 (2014).

53. T. H. Frawley, B. Muhling, S. Brodie, H. Blondin, H. Welch, M. C. Arostegui, S. J. Bograd, C. D. Braun, M. A. Cimino, N. Farchadi, E. L. Hazen, D. Tommasi, M. Jacox, Dynamic human, oceanographic, and ecological factors mediate transboundary fishery overlap across the Pacific high seas. *Fish Fish.* **25**, 60–81 (2024).

54. B. Worm, S. Orofino, E. S. Burns, N. G. D'Costa, L. Manir Feitosa, M. L. D. Palomares, L. Schiller, D. Bradley, Global shark fishing mortality still rising despite widespread regulatory change. *Science* **383**, 225–230 (2024).

55. D. J. McCauley, P. Woods, B. Sullivan, B. Bergman, C. Jablonicky, A. Roan, M. Hirshfield, K. Boerder, B. Worm, Ending hide and seek at sea. *Science* **351**, 1148–1150 (2016).

56. K. L. Seto, N. A. Miller, D. Kroodsma, Q. Hanich, M. Miyahara, R. Saito, K. Boerder, M. Tsuda, Y. Oozeki, O. Urrutia S., Fishing through the cracks: The unregulated nature of global squid fisheries. *Sci. Adv.* **9**, eadd8125 (2023).

57. M. A. Cimino, M. Anderson, T. Schramek, S. Merrifield, E. J. Terrill, Towards a Fishing Pressure Prediction System for a Western Pacific EEZ. *Sci. Rep.* **9**, 461 (2019).

58. H. Young, J. W. Akana Murphy, R. W. Baird, D. Benavente, A. Friedlander, Y. Golbuu, M. Hixon, S. Kaho'ohalahala, K. Kikiloi, B. R. C. Kennedy, D. McCauley, L. Morgan, R. Richmond, R. Rotjan, S. Shaffer, E. Sala, G. Sancho, S. Maxwell, *Expansion of the Pacific Remote Islands Marine National Monument: Honoring Cultural and Biological Legacies* (2022).

59. M. Carrere, "Barco chino que pescaba ilegalmente en Argentina tendría un 'barco gemelo' para cometer ilegalidades", *Mongabay*, 11 May 2020; https://es.mongabay.com/2020/05/oceanospesca-ilegal-en-argentina/.

60. M. Jordan, Spain Sanctions Vessels for Disabling Tracking Devices Following Eye-Opening Oceana Report (Oceana, 2023); https://oceana.org/press-releases/spain-sanctions-vessels-fordisabling-tracking-devices-following-eye-opening-oceana-report/.

61. J. Park, J. Van Osdel, J. Turner, C. M. Farthing, N. A. Miller, H. L. Linder, G. Ortuño Crespo, G. Carmine, D. A. Kroodsma, Tracking elusive and shifting identities of the global fishing fleet. *Sci. Adv.* **9**, eabp8200 (2023).

62. M. G. Jacox, M. A. Alexander, S. J. Bograd, J. D. Scott, Thermal displacement by marine heatwaves. *Nature* **584**, 82–86 (2020).

63. B. L. Clark, A. P. B. Carneiro, E. J. Pearmain, M.-M. Rouyer, T. A. Clay, W. Cowger, R. A.

Phillips, *A. Manica*, C. Hazin, M. Eriksen, J. González-Solís, J. Adams, Y. V. Albores-Barajas, J. Alfaro-Shigueto, M. S. Alho, D. T. Araujo, J. M. Arcos, J. P. Y. Arnould, N. J. P. Barbosa, C. Barbraud, A. M. Beard, J. Beck, E. A. Bell, D. G. Bennet, M. Berlincourt, M. Biscoito, O. K. Bjørnstad, M. Bolton, K. A. Booth Jones, J. J. Borg, K. Bourgeois, V. Bretagnolle, J. Bried, J. V. Briskie, M. De L. Brooke, K. C. Brownlie, L. Bugoni, L. Calabrese, L. Campioni, M. J. Carey, R. D. Carle, N. Carlile, A. R. Carreiro, P. Catry, T. Catry, J. G. Cecere, F. R. Ceia, Y. Cherel, C.-Y. Choi, M. Cianchetti-Benedetti, R. H. Clarke, J. B. Cleeland, V. Colodro, B. C. Congdon, J. Danielsen, F. De Pascalis, Z. Deakin, N. Dehnhard, G. Dell'Omo, K. Delord, S. Descamps, B. J. Dilley, H. A. Dinis, J. Dubos, B. J. Dunphy, L. M. Emmerson, A. I. Fagundes, A. L. Fayet, J. J. Felis, J. H. Fischer, A. N. D. Freeman, A. Fromant, G. Gaibani, D. García, C. Gjerdrum, I. S. G. C. Gomes, M. G. Forero, J. P. Granadeiro, W. J. Grecian, D. Grémillet, T. Guilford, G. T. Hallgrimsson, L. R. Halpin, E. S. Hansen, A. Hedd, M. Helberg, H. H. Helgason, L. M. Henry, H. F. R. Hereward, M. Hernandez-Montero, M. A. Hindell, P. J. Hodum, S. Imperio, A. Jaeger, M. Jessopp, P. G. R. Jodice, C. G. Jones, C. W. Jones, J. E. Jónsson, A. Kane, S. Kapelj, Y. Kim, H. Kirk, Y. Kolbeinsson, P. L. Kraemer, L. Krüger, P. Lago, T. J. Landers, J. L. Lavers, M. Le Corre, A. Leal, M. Louzao, J. Madeiros, M. Magalhães, M. L. Mallory, J. F. Masello, B. Massa, S. Matsumoto, F. McDuie, L. McFarlane Tranquilla, F. Medrano, B. J. Metzger, T. Militão, W. A. Montevecchi, R. C. Montone, L. Navarro-Herrero, V. C. Neves, D. G. Nicholls, M. A. C. Nicoll, K. Norris, S. Oppel, D. Oro, E. Owen, O. Padget, V. H. Paiva, *D. Pala*, J. M. Pereira, C. Péron, M. V. Petry, A. de Pina, A. T. M. Pina, P. Pinet, P. A. Pistorius, I. L. Pollet, B. J. Porter, T. A. Poupart, C. D. L. Powell, C. B. Proaño, J. Pujol-Casado, P. Quillfeldt, J. L. Quinn, A. F. Raine, H. Raine, I. Ramírez, J. A. Ramos, R. Ramos, A. Ravache, M. J. Rayner, T. A. Reid, G. J. Robertson, G. J. Rocamora, D. P. Rollinson, R. A. Ronconi, A. Rotger, D. Rubolini, K.

Ruhomaun, A. Ruiz, J. C. Russell, P. G. Ryan, S. Saldanha, A. Sanz-Aguilar, M. Sardà-Serra, Y. G. Satgé, K. Sato, W. C. Schäfer, S. Schoombie, S. A. Shaffer, N. Shah, A. Shoji, D. Shutler, I. A. Sigurðsson, M. C. Silva, A. E. Small, C. Soldatini, H. Strøm, C. A. Surman, A. Takahashi, V. R. V. Tatayah, G. A. Taylor, R. J. Thomas, D. R. Thompson, P. M. Thompson, T. L. Thórarinsson, D. Vicente-Sastre, E. Vidal, E. D. Wakefield, S. M. Waugh, H. Weimerskirch, H. U. Wittmer, T. Yamamoto, K. Yoda, C. B. Zavalaga, F. J. Zino, M. P. Dias, Global assessment of marine plastic exposure risk for oceanic birds. *Nat. Commun.* **14**, 3665 (2023).

64. B. P. Wallace, R. L. Lewison, S. L. McDonald, R. K. McDonald, C. Y. Kot, S. Kelez, R. K. Bjorkland, E. M. Finkbeiner, S. Helmbrecht, L. B. Crowder, Global patterns of marine turtle bycatch. *Conserv. Lett.* **3**, 131–142 (2010).

65. NLA International, "Proposed model for Seabed 2030—Seabed Mapping Benefits Analysis and Prioritization" (NLA International, 2022); https://seabed2030.org/wpcontent/uploads/2023/06/20220617-Proposed-model-for-Seabed-2030-Benefits-Analysis-and-Prioritisation_Final-2.pdf.

66. J. Elith, J. R. Leathwick, T. Hastie, A working guide to boosted regression trees. *J. Anim. Ecol.* **77**, 802–813 (2008).

67. Flanders Marine Institute, Union of the ESRI Country shapefile and the Exclusive Economic Zones (version 3) (2020); https://doi.org/10.14284/403.

68. U.S. Coast Guard, Federal requirements for commercial fishing industry vessels (U.S. Coast Guard, 2020); www.dco.uscg.mil/Portals/9/Fed_Regs.pdf.

69. ISSF, ISSF 2023–12: Status of the World Fisheries for Tuna (ISSF, 2023); www.issfoundation.org/downloads/35754/?tmstv=1705007922.

70. J. V. Carretta, K. A. Forney, E. M. Oleson, D. W. Weller, A. R. Lang, J. D. Barker, M. Muto,

B. Hanson, A. J. Orr, H. R. Huber, M. S. Lowry, J. Barlow, J. E. Moore, D. Lynch, L. Carswell, Brownell, U.S. Pacific Marine Mamal Stock Assessments: 2018 (NOAA, 2019); https://doi.org/10.25923/x17q-2p43.

71. International Scientific Committee for Tuna and Tuna-like species in the north Pacific Ocean (ISC), Stock assessment and future projections of Blue Shark in the north Pacific Ocean through 2015. Report of the Shark Working Group (WCPFC-SC13-2017/SA-WP-10), paper presented at Western Central Pacific Fisheries Commission Scientific Committee Thirteenth Regular Session, Rarotonga, Cook Islands, 9 to 17 August 2017; www.wcpfc.int/doc/sc13-sa-wp-10/north-pacificblue-shark-assessment-placeholder.

72. International Scientific Committee for Tuna and Tuna-Like Species in the North Pacific Ocean (ISC). 2018, Stock Assessment of Shortfin Mako Shark in the North Pacific Ocean Through 2016 (WCPFC-NC14–2018/IP-06), paper presented at Western and Central Pacific Fisheries Commission, Northern Committee Fourteenth Regular Session, Fukuoka, Japan, 4 to 7 September, 2018; https://isc.fra.go.jp/pdf/ISC18/ISC_18_ANNEX_15_Shortfin_Mako_Shark_Stock_Assessment_FINAL.pdf.

73. H. Dewar, T. Eguchi, J. Hyde, D. H. Kinzey, S. Kohin, J. Moore, B. L. Taylor, R. Vetter, Status review of the northeastern Pacific population of white sharks (Carcharodon carcharias) under the Endangered Species Act (NOAA, 2013); https://repository.library.noaa.gov/view/noaa/17705.

74. J. A. Arata, P. R. Sievert, M. B. Naughton, “Status assessment of Laysan and black-footed albatrosses, North Pacific Ocean, 1923–2005” (U.S. Geological Survey Scientific Investigations Report 2009-5131, 2009); https://pubs.usgs.gov/sir/2009/5131/pdf/sir20095131.pdf.

75. National Marine Fisheries Service and U.S. Fish and Wildlife Service, “Endangered Species

Act status review of the leatherback turtle (*Dermochelys coriacea*). Report to the National

Marine Fisheries
